# Correction: Evaluating the associations and predictive performance of triglyceride-glucose index and related indicators for chronic diseases in a Chinese cohort

**DOI:** 10.1371/journal.pone.0338914

**Published:** 2025-12-12

**Authors:** The PLOS One Staff

There are errors in the author affiliations. The correct affiliations are as follows:

Hongli Liu^1^, Xinmei Mao^1^, Xuechen Wang^1^, Dan Xu^2^, Ting Wen^1^, Jipeng Li^3^

1 Outpatient Department (OPD), The General Hospital of Western Theater Command, Chengdu, China, 2 Department of UItrasound, The General Hospital of Western Theater Command, Chengdu, China, 3 Department of Radiology, China MCC5 Group Corp.Ltd.Hospital, Chengdu, China.

The publisher apologizes for the error.
