# Supplementary figures and images for: Evaluating the associations and predictive performance of triglyceride-glucose index and related indicators for chronic diseases in a Chinese cohort
Source: PLoS One. 2025 Aug 26;20(8):e0330711. doi: 10.1371/journal.pone.0330711 (PMC12380276; doi:10.1371/journal.pone.0330711)

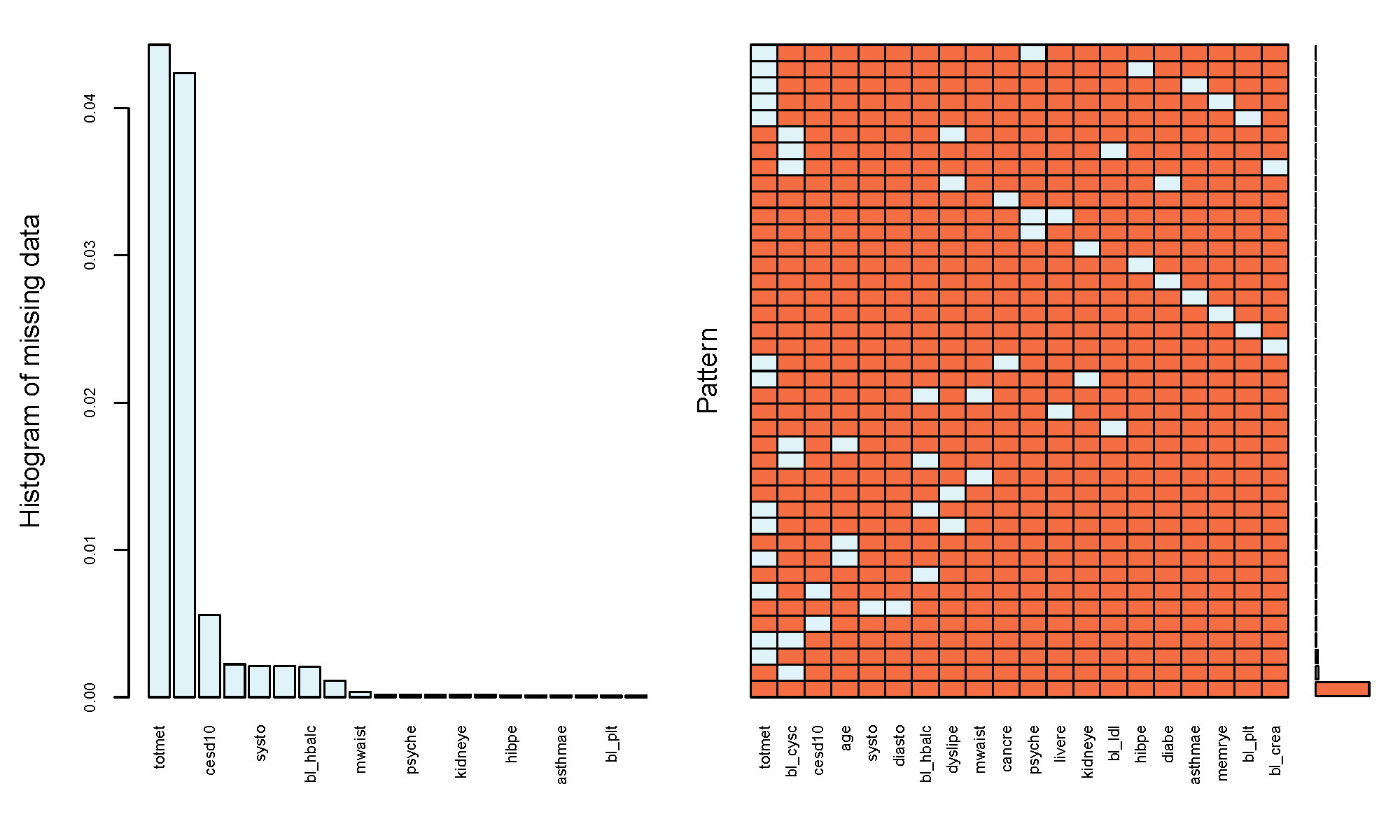

Supplement: S1 Fig — (TIF) [file pone.0330711.s001.tif]
