# Supplementary material for: Evaluating the associations and predictive performance of triglyceride-glucose index and related indicators for chronic diseases in a Chinese cohort
Source: PLoS One. 2025 Aug 26;20(8):e0330711. doi: 10.1371/journal.pone.0330711 (PMC12380276; doi:10.1371/journal.pone.0330711)
Supplement: S1 Table — (DOCX) [file pone.0330711.s002.docx]

1. **Proportional Hazards Assumption**

| Predictor | Disease | Test Statistic | P Value | PH Assumption Met |
| --- | --- | --- | --- | --- |
| TyG_WC | Diabetes | 41.561 | 0 | No |
| TyG_WC | Digestive Disease | 0.731 | 0.3925 | Yes |
| TyG_WC | Dyslipidemia | 69.58 | 0 | No |
| TyG_WC | Cardiovascular Disease | 15.637 | 1e-04 | No |
| TyG_WC | Hypertension | 19.697 | 0 | No |
| TyG_WC | Stroke | 2.099 | 0.1474 | Yes |
| TyG_WHtR | Diabetes | 7.157 | 0.0075 | No |
| TyG_WHtR | Digestive Disease | 0.758 | 0.384 | Yes |
| TyG_WHtR | Dyslipidemia | 12.566 | 4e-04 | No |
| TyG_WHtR | Cardiovascular Disease | 3.983 | 0.046 | No |
| TyG_WHtR | Hypertension | 0.393 | 0.5307 | Yes |
| TyG_WHtR | Stroke | 0.168 | 0.6819 | Yes |
| WC | Diabetes | 18.818 | 0 | No |
| WC | Digestive Disease | 0.17 | 0.6805 | Yes |
| WC | Dyslipidemia | 68.94 | 0 | No |
| WC | Cardiovascular Disease | 12.702 | 4e-04 | No |
| WC | Hypertension | 33.613 | 0 | No |
| WC | Stroke | 1.262 | 0.2613 | Yes |
| WHtR | Diabetes | 2.979 | 0.0843 | Yes |
| WHtR | Digestive Disease | 0.238 | 0.6259 | Yes |
| WHtR | Dyslipidemia | 9.042 | 0.0026 | No |
| WHtR | Cardiovascular Disease | 2.285 | 0.1306 | Yes |
| WHtR | Hypertension | 0.372 | 0.5419 | Yes |
| WHtR | Stroke | 0.014 | 0.9073 | Yes |
| BMI | Diabetes | 0.129 | 0.7191 | Yes |
| BMI | Digestive Disease | 4.638 | 0.0313 | No |
| BMI | Dyslipidemia | 1.09 | 0.2965 | Yes |
| BMI | Cardiovascular Disease | 0.208 | 0.6485 | Yes |
| BMI | Hypertension | 0.009 | 0.9227 | Yes |
| BMI | Stroke | 0.118 | 0.7307 | Yes |
| tyg | Diabetes | 47.011 | 0 | No |
| TyG | Digestive Disease | 1.591 | 0.2071 | Yes |
| TyG | Dyslipidemia | 31.815 | 0 | No |
| TyG | Cardiovascular Disease | 8.863 | 0.0029 | No |
| TyG | Hypertension | 1.822 | 0.1771 | Yes |
| TyG | Stroke | 1.522 | 0.2173 | Yes |
| TyG_BMI | Diabetes | 0.004 | 0.9499 | Yes |
| TyG_BMI | Digestive Disease | 3.881 | 0.0488 | No |
| TyG_BMI | Dyslipidemia | 0.932 | 0.3344 | Yes |
| TyG_BMI | Cardiovasculr Disease | 1.545 | 0.2139 | Yes |
| TyG_BMI | Hypertension | 0.07 | 0.7906 | Yes |
| TyG_BMI | Stroke | 0.029 | 0.8658 | Yes |

TyG, triglyceride-glucose; BMI, body mass index; WC, waist circumference; WHtR, waist-to-height ratio.

Yes: The proportional hazards assumption is met. The effect of this predictor on hazard is constant over time.

NO:The proportional hazards assumption is violated. Consider time-dependent covariate or stratification.
