# Supplementary material for: Evaluating the associations and predictive performance of triglyceride-glucose index and related indicators for chronic diseases in a Chinese cohort
Source: PLoS One. 2025 Aug 26;20(8):e0330711. doi: 10.1371/journal.pone.0330711 (PMC12380276; doi:10.1371/journal.pone.0330711)
Supplement: S2 Table — (DOCX) [file pone.0330711.s003.docx]

**Table S2. Model Calibration and Discrimination**

| Predictor | Disease | C Index  (95% CI) | Calibration Slope | Calibration Intercept | Calibration Assessment | Discrimination Assessment |
| --- | --- | --- | --- | --- | --- | --- |
| TyG | Hypertension | 0.633 (0.625-0.64) | 0.287 | 0.14 | Poor calibration (p <= 0.05) | Acceptable discrimination (0.6 <= C < 0.7) |
| TyG | Diabetes | 0.723 (0.716-0.73) | 0.109 | 0.028 | Poor calibration (p <= 0.05) | Good discrimination  (0.7 <= C < 0.8) |
| TyG | Cardiovascular Disease | 0.616 (0.608-0.623) | 0.25 | 0.038 | Poor calibration (p <= 0.05) | Acceptable discrimination (0.6 <= C < 0.7) |
| TyG | Stroke | 0.643 (0.635-0.65) | 0.065 | 0.006 | Poor calibration (p <= 0.05) | Acceptable discrimination (0.6 <= C < 0.7) |
| TyG | Dyslipidemia | 0.66 (0.652-0.667) | 0.228 | 0.067 | Poor calibration (p <= 0.05) | Acceptable discrimination (0.6 <= C < 0.7) |
| TyG | Digestive Disease | 0.548 (0.54-0.555) | 0.256 | 0.075 | Poor calibration (p <= 0.05) | Poor discrimination  (C < 0.6) |
| TyG_BMI | Hypertension | 0.607 (0.6-0.615) | 0.35 | 0.107 | Poor calibration (p <= 0.05) | Acceptable discrimination (0.6 <= C < 0.7) |
| TyG_BMI | Diabetes | 0.588 (0.58-0.595) | 0.191 | -0.001 | Poor calibration (p <= 0.05) | Poor discrimination  (C < 0.6) |
| TyG_BMI | Cardiovascular Disease | 0.61 (0.602-0.617) | 0.265 | 0.032 | Poor calibration (p <= 0.05) | Acceptable discrimination (0.6 <= C < 0.7) |
| TyG_BMI | Stroke | 0.624 (0.617-0.632) | 0.072 | 0.005 | Poor calibration (p <= 0.05) | Acceptable discrimination (0.6 <= C < 0.7) |
| TyG_BMI | Dyslipidemia | 0.598 (0.591-0.606) | 0.269 | 0.064 | Poor calibration (p <= 0.05) | Poor discrimination  (C < 0.6) |
| TyG_BMI | Digestive Disease | 0.546 (0.538-0.554) | 0.265 | 0.065 | Poor calibration (p <= 0.05) | Poor discrimination  (C < 0.6) |
| WC | Hypertension | 0.664 (0.657-0.672) | 0.301 | 0.113 | Poor calibration (p <= 0.05) | Acceptable discrimination (0.6 <= C < 0.7) |
| WC | Diabetes | 0.659 (0.652-0.667) | 0.178 | -0.012 | Poor calibration (p <= 0.05) | Acceptable discrimination (0.6 <= C < 0.7) |
| WC | Cardiovascular Disease | 0.629 (0.621-0.636) | 0.25 | 0.032 | Poor calibration (p <= 0.05) | Acceptable discrimination (0.6 <= C < 0.7) |
| WC | Stroke | 0.642 (0.635-0.649) | 0.068 | 0.002 | Poor calibration (p <= 0.05) | Acceptable discrimination (0.6 <= C < 0.7) |
| WC | Dyslipidemia | 0.661 (0.654-0.669) | 0.251 | 0.049 | Poor calibration (p <= 0.05) | Acceptable discrimination (0.6 <= C < 0.7) |
| WC | Digestive Disease | 0.56 (0.552-0.568) | 0.245 | 0.086 | Poor calibration (p <= 0.05) | Poor discrimination  (C < 0.6) |
| TyG_WC | Hypertension | 0.668 (0.661-0.676) | 0.279 | 0.127 | Poor calibration (p <= 0.05) | Acceptable discrimination (0.6 <= C < 0.7) |
| TyG_WC | Diabetes | 0.714 (0.707-0.721) | 0.133 | 0.007 | Poor calibration (p <= 0.05) | Good discrimination  (0.7 <= C < 0.8) |
| TyG_WC | Cardiovascular Disease | 0.63 (0.622-0.637) | 0.241 | 0.038 | Poor calibration (p <= 0.05) | Acceptable discrimination (0.6 <= C < 0.7) |
| TyG_WC | Stroke | 0.651 (0.643-0.658) | 0.063 | 0.005 | Poor calibration (p <= 0.05) | Acceptable discrimination (0.6 <= C < 0.7) |
| TyG_WC | Dyslipidemia | 0.682 (0.675-0.689) | 0.222 | 0.06 | Poor calibration (p <= 0.05) | Acceptable discrimination (0.6 <= C < 0.7) |
| TyG_WC | Digestive Disease | 0.558 (0.55-0.566) | 0.244 | 0.089 | Poor calibration (p <= 0.05) | Poor discrimination  (C < 0.6) |
| WHtR | Hypertension | 0.635 (0.627-0.642) | 0.372 | 0.067 | Poor calibration (p <= 0.05) | Acceptable discrimination (0.6 <= C < 0.7) |
| WHtR | Diabetes | 0.617 (0.609-0.624) | 0.22 | -0.034 | Poor calibration (p <= 0.05) | Acceptable discrimination (0.6 <= C < 0.7) |
| WHtR | Cardiovascular Disease | 0.618 (0.61-0.625) | 0.259 | 0.029 | Poor calibration (p <= 0.05) | Acceptable discrimination (0.6 <= C < 0.7) |
| WHtR | Stroke | 0.635 (0.628-0.643) | 0.072 | 0.001 | Poor calibration (p <= 0.05) | Acceptable discrimination (0.6 <= C < 0.7) |
| WHtR | Dyslipidemia | 0.623 (0.616-0.631) | 0.307 | 0.023 | Poor calibration (p <= 0.05) | Acceptable discrimination (0.6 <= C < 0.7) |
| WHtR | Digestive Disease | 0.556 (0.549-0.564) | 0.272 | 0.065 | Poor calibration (p <= 0.05) | Poor discrimination  (C < 0.6) |
| TyG_WHtR | Hypertension | 0.647 (0.64-0.655) | 0.373 | 0.06 | Poor calibration (p <= 0.05) | Acceptable discrimination (0.6 <= C < 0.7) |
| TyG_WHtR | Diabetes | 0.671 (0.664-0.678) | 0.276 | -0.087 | Poor calibration (p <= 0.05) | Acceptable discrimination (0.6 <= C < 0.7) |
| TyG_WHtR | Cardiovascular Disease | 0.622 (0.614-0.629) | 0.259 | 0.027 | Poor calibration (p <= 0.05) | Acceptable discrimination (0.6 <= C < 0.7) |
| TyG_WHtR | Stroke | 0.644 (0.637-0.651) | 0.073 | -0.001 | Poor calibration (p <= 0.05) | Acceptable discrimination (0.6 <= C < 0.7) |
| TyG_WHtR | Dyslipidemia | 0.647 (0.639-0.654) | 0.335 | -0.004 | Poor calibration (p <= 0.05) | Acceptable discrimination (0.6 <= C < 0.7) |
| TyG_WHtR | Digestive Disease | 0.555 (0.548-0.563) | 0.266 | 0.071 | Poor calibration (p <= 0.05) | Poor discrimination  (C < 0.6) |
| BMI | Hypertension | 0.605 (0.597-0.612) | 0.346 | 0.112 | Poor calibration (p <= 0.05) | Acceptable discrimination (0.6 <= C < 0.7) |
| BMI | Diabetes | 0.579 (0.571-0.586) | 0.169 | 0.017 | Poor calibration (p <= 0.05) | Poor discrimination  (C < 0.6) |
| BMI | Cardiovascular Disease | 0.609 (0.601-0.616) | 0.264 | 0.033 | Poor calibration (p <= 0.05) | Acceptable discrimination (0.6 <= C < 0.7) |
| BMI | Stroke | 0.623 (0.616-0.631) | 0.072 | 0.005 | Poor calibration (p <= 0.05) | Acceptable discrimination (0.6 <= C < 0.7) |
| BMI | Dyslipidemia | 0.593 (0.585-0.6) | 0.261 | 0.071 | Poor calibration (p <= 0.05) | Poor discrimination  (C < 0.6) |
| BMI | Digestive Disease | 0.546 (0.538-0.554) | 0.265 | 0.065 | Poor calibration (p <= 0.05) | Poor discrimination  (C < 0.6) |

TyG, triglyceride-glucose; BMI, body mass index; WC, waist circumference; WHtR, waist-to-height ratio.
