# Supplementary material for: Evaluating the associations and predictive performance of triglyceride-glucose index and related indicators for chronic diseases in a Chinese cohort
Source: PLoS One. 2025 Aug 26;20(8):e0330711. doi: 10.1371/journal.pone.0330711 (PMC12380276; doi:10.1371/journal.pone.0330711)
Supplement: Attachment — Submitted filename: PONE-D-25-03753.pdf [file pone.0330711.s005.pdf]

PONE-D-25-03753

by Debasis Mitra

Submission date: 08-Jun-2025 01:13PM (UTC+0530)
Submission ID: 2694354990
File name: manuscript.docx (468.06K)
Word count: 10397
Character count: 62836

Evaluating the Associations and Predictive Performance of Triglyceride-Glucose Index and Related Indicators for Chronic Diseases in a Chinese Cohort

Hongli Liu[1#], Xinmei Mao[1#], Xuechen Wang[1#], Dan Xu[3], Ting Wen[1], Jipeng Li[2*]

1 Outpatient Department(OPD), The General Hospital of Western Theater Command， Chengdu 610083, China

2 Department of Radiology, China MCC5 Group Corp.Ltd.Hospital, Chengdu 610081, China

3 Department of Ultrasound, The General Hospital of Western Theater Command, Chengdu 610083, China

* Authors to whom correspondence should be addressed.

# These authors contributed equally to this work.

E-**mail addresses:** LHL_808@outlook.com (Hongli Liu), 3709042784@sohu.com (Xinmei Mao), WXC_812@outlook.com (Xuechen Wang), Xd_819@outlook.com (Dan Xu), 15208353500@163.com (Ting Wen), Ljp_712@outlook.com (Jipeng Li)

Abstract

Background Triglyceride and glucose (TyG) indices have been used as predictors of several chronic diseases. However, there is currently a lack of research that can comprehensively reflect the impact of TyG-related indicators on chronic diseases in middle-aged and elderly populations. The aim of this study was to investigate the relationship of TyG and its related

indicators with chronic diseases and their time-dependent predictive ability in the elderly.

Study design Retrospective observational cohort study using China Health and Retirement Longitudinal Study (CHARLS) 2011-2020 data.

Methods Based on longitudinal data obtained from the CHARLS from 2011 to 2020, a total of 12,966 participants were included in the study. Participants were stratified into three groups according to their TyG index. Pearson correlation coefficient and Cox model are used to assess the relationship between the TyG index, its parameters, and common chronic diseases, while Harrell's C-index is used to evaluate their risk prediction capability.

Results The TyG index and its related indicators exhibit a positive dose-response relationship with the risk of diabetes, heart disease, dyslipidemia, hypertension, and stroke, while demonstrating a negative dose-response relationship with digestive system diseases. Harrell's C-index results indicated that TyG-WC demonstrates superior predictive performance overall.

Conclusion The TyG index and its related indicators are significantly correlated with newly onset emerging chronic diseases, with TyG-WC exhibits superior risk prediction performance.

Keywords: TyG index; TygG-WC; Chronic disease; Dose-response relationship; Time-dependent risk prediction capability

Introduction

Chronic diseases, defined as conditions lasting a year or more that require ongoing medical attention or limit daily activities, include heart disease, cancer, diabetes, depression, and other serious illnesses[1]. As the largest population in the world, China is facing significant challenges due to its rapidly aging population, which has become a pressing social issue[2]. One of the most critical consequences of this demographic shift is the sustained increase in the prevalence of chronic diseases[3]. By 2040, the number of individuals aged 60 and above in China is projected to reach 402 million, accounting for 28% of the total population, with more than 75% of them expected to suffer from at least one chronic disease[4]. Moreover, the risk of developing multiple chronic conditions rises significantly with age[5]. Therefore, effective prevention and management of chronic diseases in the elderly are crucial for alleviating the health, social, psychological, and economic burdens associated with these conditions.

Insulin plays a key role in the pathogenesis of various chronic diseases by influencing blood glucose levels, cell division, and the metabolism of fats and proteins[6]. Under normal physiological conditions, elevated plasma glucose levels can lead to increased insulin secretion and circulating insulin levels, thereby stimulating glucose transfer to peripheral tissues and

inhibiting liver glucose production[7]. However, when insulin function is impaired, it results in a pathological state known as "insulin resistance" (IR), characterized by reduced insulin-mediated glucose control, diminished glucose utilization, abnormal lipid accumulation, and heightened lipid degradation in adipocytes[8]. IR is clinically associated with several metabolic and chronic diseases, including obesity, type 2 diabetes and its complications, non-alcoholic fatty liver disease, tumors, and cardiovascular disease[8],[9]. Furthermore, cellular senescence, often observed in age-related chronic diseases, is believed to be linked to IR, with both conditions mutually reinforcing each other[10]. Addressing IR is therefore essential for preventing and managing these chronic diseases.

Despite its importance, the relationship between IR and chronic diseases has not been fully elucidated and remains a topic of ongoing debate. Previous studies have utilized the homeostasis model assessment (HOMA) as a relatively simple and reliable method for assessing IR[11]. More recently, the Triglyceride-Glucose (TyG) index, an IR marker derived from fasting triglyceride and blood glucose levels, has become a simple and reliable alternative to HOMA[12]-[14]. The TyG index is now widely used to assess the association between IR and chronic diseases, including chronic kidney disease[15], chronic lung disease[16], osteoarthritis[17], and non-alcoholic fatty liver disease[18]. Evidence suggests that a higher TyG index

is associated with an increased risk of chronic disease onset[19]-[21]. In addition to the effects of metabolic biomarkers such as triglycerides and glucose, disorders of lipid metabolism also play an important role in the adverse consequences caused by IR. Recent studies have shown that combining the TyG index with obesity parameters (such as body mass index [BMI], waist circumference [WC], and waist-to-height ratio [WHtR]) can enhance the identification of IR. Modified TyG indices that integrate these two groups of parameters have also been applied to various diseases[22]-[24]. Surveys on chronic diseases in the elderly also have consistently recognized the TyG index and related parameters as valuable predictive indicators[20],[25],[26]. Maintaining a healthy index can help prevent or delay the development of chronic diseases, but the specific association needs to be investigated, because too high or too low can lead to an increased risk of disease. Therefore, our study aims to investigate the association between TyG and its related parameters and chronic diseases in middle-aged and elderly people in China using data from the China Health and Retirement Longitudinal Study (CHARLS) database from 2011 to 2020.

Data and methods

Data Source and Preprocessing

Data were obtained from the China Health and Retirement Longitudinal

Study (CHARLS), a nationally representative survey of middle-aged and elderly Chinese individuals aged 45 and above. The CHARLS study was approved by the Institutional Review Board of Peking University (IRB00001052-11015). We filtered the data for non-missing TyG and TyG-BMI values and variables with ≤5% missing data. Multiple imputation was performed using the mice package in R, creating a prediction matrix and specifying imputation methods based on variable types. Outliers in continuous variables were removed using a function based on the normal distribution and 3σ criterion (Figure S1).

Baseline Characteristics and Correlation Analysis

Participants were divided into three groups based on TyG values: Group 1 (TyG < 8.36), Group 2 (8.36 ≤ TyG < 8.87), and Group 3 (TyG ≥ 8.87). The TyG index was calculated as Ln (triacylglycerol (mg/dL) × fasting blood glucose (mg/dL)/2). Baseline characteristics were summarized using the gtsummary package in R, presenting categorical variables as counts and percentages, and continuous variables as means and standard deviations. We calculated obesity-related scores including WC, WHtR, TyG-WC, and TyG-WHtR. Correlation analysis between various diseases and these scores was performed using the phi coefficient for categorical variables and Pearson correlation for continuous variables. Results were visualized using the corrplot package.

Dose-Response Relationship Analysis

We used data from the CHARLS to calculate the onset time for each disease in the disease list (hibpe, diabe, hearte, stroke, dyslipe, and digeste). For each individual, we determined if the disease was present at the baseline survey. If not, we found the first survey where the disease was reported and calculated the time difference in years and months from the baseline survey. If the disease never occurred, we used the last survey's year and month. The calculated disease onset times were then added to the processed data frame.

To investigate the dose-response relationships between various predictors (TyG, BMI, TyG-BMI, WC, TyG-WC, WHtR, and TyG-WHtR) and diseases, we used restricted cubic spline (RCS) analysis. For each predictor-disease combination, we fitted a Cox regression model adjusting for relevant covariates (age, gender, rural, edu, marry, smokev, drinkev, and conditionally hibpe and diabe). We then fitted an RCS model with 3 knots using the rms package's cph() function. We created prediction plots using the Predict function, setting the reference point to the median of the predictor variable. The predicted hazard ratios and their 95% confidence intervals were plotted using ggplot2, with the median as the reference line.

Cox Proportional Hazards Model Analysis

Univariate Cox proportional hazards regression analysis was conducted to evaluate the impact of predictive factors on chronic disease risk. The TyG index was divided into tertiles. Four models with increasing levels of adjustment were used: Model 1 adjusted for age and gender; Model 2 additionally adjusted for marital status, residential area, education level, smoking status, and alcohol consumption. To address potential residual confounding, we conducted several sensitivity analyses with additional adjustments when data were available. Hazard ratio, 95% confidence interval (CI), and p-value were estimated for each predictor-disease combination.

Since the fundamental assumption of the Cox model is the proportional hazards assumption—that is, the influence of each predictor on the outcome variable remains relatively constant throughout the observation period—we tested this assumption for all models. Specifically, for each combination of predictor variables (TyG, TyG-BMI, WC, TyG-WC, WHtR, TyG-WHR, and BMI) and six chronic diseases (hypertension, diabetes, cardiovascular disease, stroke, dyslipidemia, and digestive system diseases), we used the standard test based on Schoenfeld residuals for evaluation. This method assesses whether the effect of a predictor changes over time by testing the correlation between residuals and time; a p-value

greater than 0.05 indicates that the proportional hazards assumption is satisfied.

For predictor-disease combinations that violated the proportional hazards assumption, we incorporated interaction terms between predictors and time (time-dependent covariates) into the models to adjust for time-varying effects. This approach allows hazard ratios of predictors to change over time, enabling more accurate risk estimation. For example, in the model of the relationship between TyG-WC and diabetes, which significantly violated the proportional hazards assumption (P<0.001), we added an interaction term between TyG-WC and time to reflect its time-varying effect.

Assessment of Model Calibration and Discriminative Ability

In terms of risk prediction capability assessment, we used Harrell's C-index, which measures a model's ability to distinguish between high-risk and low-risk individuals. The index ranges from 0.5 (no predictive ability) to 1.0 (perfect prediction).

To comprehensively evaluate the performance of predictive models, we adopted a two-pronged assessment strategy: discrimination and calibration. Discrimination assessment was primarily measured by Harrell's C-index

and its 95% CI, which evaluates the model's ability to distinguish between individuals with and without the event. The C-index ranges from 0.5 to 1.0, where 0.5 indicates no predictive ability (equivalent to random guessing), and 1.0 indicates perfect prediction. We defined a C-index ≥ 0.8 as "excellent discrimination", 0.7 ≤ C < 0.8 as "good discrimination", 0.6≤ C < 0.7 as "acceptable discrimination", and C < 0.6 as "poor discrimination".

Model calibration was evaluated using two methods: (1) Calibration slope and intercept, derived by dividing predicted risks into deciles and calculating the linear regression between the average predicted probability and the observed event rate in each risk group. An ideal calibration slope should be close to 1.0, and the intercept close to 0, indicating high consistency between predicted probabilities and observed event rates. (2) Hosmer-Lemeshow test, used to assess the goodness-of-fit between predicted probabilities and observed events, where p>0.05 indicates good model calibration. All calibration analyses were performed at the reference follow-up time point (the median follow-up time for each disease).

Results

Characteristics of the study population

In this study, a total of 12,966 participants were recruited and divided into three groups based on the distribution of the TyG index, ranging from low to high, the average values

were 8.05, 8.61, and 9.28 respectively (Table 1). From the perspective of social demographic characteristics, the average ages of Group 1 to Group 3 are 64.60, 65.00, and 64.81, respectively. The majority of them are urban residents, but the proportion gradually decreases, while the proportion of males gradually increases. In addition, the educational level of the study population is generally low, as more than 60% of the participants have no formal education or only completed primary or secondary education. In terms of marital status, more than 80% of the participants are married. From the perspective of health indicators and biochemical parameters, as the TyG index increases, the proportion of males rises, along with intergroup weight, WC, BMI, and the TyG BMI index. Most participants had no history of diabetes or other chronic diseases in Group 1; however, in Groups 2 and 3 with higher TyG index, the prevalence of hypertension, diabetes, dyslipidemia, heart disease, stroke, arthritis, and memory impairment showed an increasing trend with rising TyG levels. In terms of biochemical parameters, the group with a higher TyG index exhibited elevated levels of white-blood-cell, blood-platelet-count, glucose, total-cholesterol, triglycerides, c-reaction-protein, glycosylated-hemoglobin, uric-acid, hematocrit-value, hemoglobin, systolic-pressure and diastolic-pressure, while mean-corpuscular-volume, blood-urea-nitrogen, high-density-lipoprotein-cholesterol and total-metabolic-output levels decreased as TyG levels increased.

Table 1. Characteristics of 12966 participants according to baseline TyG levels

Characteristic		Group 1 (n=4,428)	Group 2 (n=4,379)	Group 3 (n=4,159)
Age, years, mean (SD)		64.60 (10.34)	65.00 (9.89)	64.81 (9.46)
Gender, n (%)	Male	2,140 (48.3%)	2,496 (57.0%)	2,454 (59.0%)
	Female	2,288 (51.7%)	1,883 (43.0%)	1,705 (41.0%)
Residence, n (%)	Rural	1,423 (32.1%)	1,677 (38.3%)	1,855 (44.6%)
	Urban	3,005 (67.9%)	2,702 (61.7%)	2,304 (55.4%)
Edu, n (%)	Uneducated	2,067 (46.7%)	2,041 (46.6%)	1,844 (44.3%)
	Primary	1,041 (23.5%)	983 (22.4%)	927 (22.3%)
	Secondary	889 (20.1%)	873 (19.9%)	901 (21.7%)
	Third	431 (9.73%)	482 (11.0%)	487 (11.7%)
Marry, n (%)	No	822 (18.6%)	787 (18.0%)	739 (17.8%)
	Yes	3,606 (81.4%)	3,592 (82.0%)	3,420 (82.2%)
Smokev, n (%)	No	2,401 (54.2%)	2,630 (60.1%)	2,535 (61.0%)
	Yes	2,027 (45.8%)	1,749 (39.9%)	1,624 (39.0%)
Drinkev, n (%)	No	2,270 (51.3%)	2,480 (56.6%)	2,334 (56.1%)
	Yes	2,158 (48.7%)	1,899 (43.4%)	1,825 (43.9%)
mheight, m, mean (SD)		1.58 (0.08)	1.58 (0.08)	1.58 (0.09)
mweight, kg, mean (SD)		56.42 (9.90)	59.23 (10.73)	63.09 (11.10)
mwaist, cm, mean (SD)		82.03 (9.20)	85.79 (9.92)	90.44 (9.50)
BMI, kg/m^2, mean (SD)		22.43 (3.22)	23.72 (3.47)	25.20 (3.51)

Characteristic		Group 1 (n=4,428)	Group 2 (n=4,379)	Group 3 (n=4,159)
Hibpe, n (%)	No	3,003 (67.8%)	2,603 (59.4%)	2,026 (48.7%)
	Yes	1,425 (32.2%)	1,776 (40.6%)	2,133 (51.3%)
Diabe, n (%)	No	4,166 (94.1%)	3,894 (88.9%)	3,268 (78.6%)
	Yes	262 (5.92%)	485 (11.1%)	891 (21.4%)
Cancre, n (%)	No	4,311 (97.4%)	4,244 (96.9%)	4,036 (97.0%)
	Yes	117 (2.64%)	135 (3.08%)	123 (2.96%)
Lunge, n (%)	No	3,721 (84.0%)	3,623 (82.7%)	3,523 (84.7%)
	Yes	707 (16.0%)	756 (17.3%)	636 (15.3%)
Dyslipe, n (%)	No	3,706 (83.7%)	3,325 (75.9%)	2,620 (63.0%)
	Yes	722 (16.3%)	1,054 (24.1%)	1,539 (37.0%)
Hearte, n (%)	No	3,597 (81.2%)	3,328 (76.0%)	3,049 (73.3%)
	Yes	831 (18.8%)	1,051 (24.0%)	1,110 (26.7%)
Stroke, n (%)	No	4,147 (93.7%)	4,063 (92.8%)	3,745 (90.0%)
	Yes	281 (6.35%)	316 (7.22%)	414 (9.95%)
Psyche, n (%)	No	4,296 (97.0%)	4,250 (97.1%)	4,017 (96.6%)
	Yes	132 (2.98%)	129 (2.95%)	142 (3.41%)
Arthre, n (%)	No	2,730 (61.7%)	2,595 (59.3%)	2,414 (58.0%)
	Yes	1,698 (38.3%)	1,784 (40.7%)	1,745 (42.0%)
Livere, n (%)	No	4,109 (92.8%)	4,065 (92.8%)	3,863 (92.9%)
	Yes	319 (7.20%)	314 (7.17%)	296 (7.12%)
Kidneye, n (%)	No	3,965 (89.5%)	3,928 (89.7%)	3,695 (88.8%)
	Yes	463 (10.5%)	451 (10.3%)	464 (11.2%)
Digeste, n (%)	No	2,987 (67.5%)	2,940 (67.1%)	2,843 (68.4%)

Characteristic		Group 1 (n=4,428)	Group 2 (n=4,379)	Group 3 (n=4,159)
	Yes	1,441 (32.5%)	1,439 (32.9%)	1,316 (31.6%)
Asthma, n (%)	No	4,124 (93.1%)	4,036 (92.2%)	3,862 (92.9%)
	Yes	304 (6.87%)	343 (7.83%)	297 (7.14%)
Memrye, n (%)	No	4,212 (95.1%)	4,140 (94.5%)	3,897 (93.7%)
	Yes	216 (4.88%)	239 (5.46%)	262 (6.30%)
bl_wbc, /L, mean (SD)		5.63 (1.56)	5.93 (1.59)	6.21 (1.61)
bl_mcv, fL, mean (SD)		91.92 (6.96)	91.79 (6.60)	91.50 (6.36)
bl_plt, /L, mean (SD)		199.55 (62.63)	204.89 (63.60)	208.71 (65.78)
bl_bun, mmol/L, mean (SD)		15.61 (4.28)	15.22 (4.18)	14.96 (4.00)
bl_glu, mg/dL, mean (SD)		90.32 (12.22)	98.07 (15.01)	111.06 (23.61)
bl_crea,mg/dL, mean (SD)		0.78 (0.18)	0.78 (0.17)	0.78 (0.18)
bl_cho, mg/dL, mean (SD)		171.74 (30.39)	185.01 (32.32)	194.02 (34.07)
bl_tg, mg/dL, mean (SD)		72.16 (15.79)	114.64 (21.63)	206.80 (65.90)
bl_hdl, mg/dL, mean (SD)		55.82 (11.29)	51.34 (10.64)	46.36 (9.49)
bl_ldl, mg/dL, mean (SD)		98.73 (26.25)	109.05 (28.12)	106.49 (30.09)
bl_crp, mg/dL, mean (SD)		1.66 (2.48)	1.88 (2.47)	2.49 (2.51)
bl_hbalc, %, mean (SD)		5.57 (0.50)	5.68 (0.57)	5.90 (0.73)
bl_ua, mg/dL, mean (SD)		4.50 (1.20)	4.74 (1.27)	5.11 (1.30)
bl_hct, %, mean (SD)		40.92 (5.01)	41.46 (4.90)	41.83 (4.89)
bl_hgb, g/dL, mean (SD)		13.61 (1.73)	13.79 (1.70)	13.93 (1.70)
bl_cysc, mg/L, mean (SD)		0.84 (0.19)	0.87 (0.20)	0.87 (0.19)
Totmet, MET, mean (SD)		5,931.26 (5,740.31)	5,438.53 (5,515.70)	5,014.03 (5,223.15)

Characteristic	Group 1 (n=4,428)	Group 2 (n=4,379)	Group 3 (n=4,159)
TyG, mean (SD)	8.05 (0.23)	8.61 (0.15)	9.28 (0.32)
TyG_BMI, mean (SD)	180.58 (27.16)	203.70 (30.46)	233.83 (35.03)
systo, mmHg, mean (SD)	123.97 (19.42)	126.58 (18.98)	130.30 (18.82)
diasto, mmHg, mean (SD)	73.00 (11.11)	74.67 (10.95)	76.72 (11.03)
cesd10, scores, mean (SD)	8.90 (6.38)	8.98 (6.47)	8.68 (6.32)

Due to China's large population base and significant geographical differences, which may also influence chronic disease status, we further analyzed the differences in demographic and clinical characteristics between urban and rural residents (Table 2), as well as the distribution of chronic disease prevalence rates among participants from various provinces in China, including hypertension, diabetes, and cardiovascular diseases (Table 3). The results showed significant differences ($P < 0.05$) between urban and rural populations in sex, education level, smoking history, BMI, diabetes, pulmonary diseases, dyslipidemia, cardiovascular diseases, and osteoarthritis (Table 2). Analyses of different provinces revealed significant regional variations in the distribution of chronic diseases across China. For example, the average prevalence rates were relatively high in Beijing (38.2%), Inner Mongolia (38.5%), Tianjin (36.6%), and Hebei (35.3%), while those in Guangdong (14.7%) and Fujian (16.1%) were relatively low (Table 3). Additionally, two

geographical distribution maps were created: a map showing the distribution of participant numbers across provinces (Fig 1) and a geographic distribution map of average chronic disease prevalence rates (Fig 2). These visualizations intuitively demonstrate the national distribution characteristics of the CHARLS dataset and the geographic patterns of diseases.

Fig 1. Chornic disease prevalence by province

Fig 2. Distribution of CHARLS participants

Table 2. Baseline Characteristics of CHARLS Study Participants by Residence Type

Characteristics	Level	Overall (n=12966)	Urban (n= 4952)	Rural (n= 8014)	P-value
Age (years)		65.00 [57.00, 71.00]	64.00 [57.00, 71.00]	65.00 [57.00, 72.00]	0.060
Gender	Female	7091 (54.7)	2783 (56.2)	4308 (53.8)	0.007
	Male	5875 (45.3)	2169 (43.8)	3706 (46.2)	
Education Level	Below Primary School	5951 (45.9)	1672 (33.8)	4279 (53.4)	<0.001
	Primary School	2953 (22.8)	1107 (22.4)	1846 (23.0)	
	Middle School	2662 (20.5)	1258 (25.4)	1404 (17.5)	
	High School and Above	1400 (10.8)	915 (18.5)	485 (6.1)	
Smoking History	No	7566 (58.4)	2974 (60.1)	4592 (57.3)	0.002

Characteristics	Level	Overall (n=12966)	Urban (n= 4952)	Rural (n= 8014)	P-value
	Yes	5400 (41.6)	1978 (39.9)	3422 (42.7)	
Alcohol Consumption	No	7086 (54.7)	2673 (54.0)	4413 (55.1)	0.234
	Yes	5880 (45.3)	2279 (46.0)	3601 (44.9)	
Body Mass Index (kg/m²)		23.75 (3.58)	24.31 (3.58)	23.41 (3.54)	<0.001
Hypertension	No	7633 (58.9)	2900 (58.6)	4733 (59.1)	0.589
	Yes	5333 (41.1)	2052 (41.4)	3281 (40.9)	
Diabetes	No	11329 (87.4)	4248 (85.8)	7081 (88.4)	<0.001
	Yes	1637 (12.6)	704 (14.2)	933 (11.6)	
Cancer	No	12591 (97.1)	4794 (96.8)	7797 (97.3)	0.124
	Yes	375 (2.9)	158 (3.2)	217 (2.7)	
Pulmonary Disease	No	10867 (83.8)	4193 (84.7)	6674 (83.3)	0.039
	Yes	2099 (16.2)	759 (15.3)	1340 (16.7)	
Dyslipidemia	No	9652 (74.4)	3461 (69.9)	6191 (77.3)	<0.001
	Yes	3314 (25.6)	1491 (30.1)	1823 (22.7)	
Cardiovascular Disease	No	9975 (76.9)	3663 (74.0)	6312 (78.8)	<0.001
	Yes	2991 (23.1)	1289 (26.0)	1702 (21.2)	
Stroke	No	11956 (92.2)	4575 (92.4)	7381 (92.1)	0.578
	Yes	1010 (7.8)	377 (7.6)	633 (7.9)	
Psychiatric Disorder	No	12563 (96.9)	4793 (96.8)	7770 (97.0)	0.633
	Yes	403 (3.1)	159 (3.2)	244 (3.0)	
Arthritis	No	7739 (59.7)	3128 (63.2)	4611 (57.5)	<0.001
	Yes	5227 (40.3)	1824 (36.8)	3403 (42.5)	
Memory-related Disease	No	12249 (94.5)	4673 (94.4)	7576 (94.5)	0.712
	Yes	717 (5.5)	279 (5.6)	438 (5.5)	

Table 3. Provincial Distribution of CHARLS Study Participants and

Chronic Disease Prevalence

Province	Participants	Median Age (years)	Male (%)	Hypertension (%)	Diabetes (%)	Cardiovascular (%)	Average Prevalence (%)
Shandong	1,221	64.0	45.2	45.0	16.1	25.6	28.9
Henan	1,138	64.0	45.3	41.4	15.1	25.3	27.3
Sichuan	867	66.0	46.1	36.1	11.4	15.5	21.0
Anhui	784	66.0	46.9	45.4	14.7	22.8	27.6
Yunnan	779	63.0	47.4	37.2	6.8	11.9	18.7
Jiangxi	666	66.0	43.5	36.6	11.1	18.6	22.1
Hunan	633	65.0	43.8	40.1	11.8	24.6	25.5
Inner Mongolia	590	61.0	44.6	51.5	14.2	49.7	38.5
Hebei	569	65.0	45.9	53.1	16.5	36.2	35.3
Zhejiang	560	65.0	44.8	41.4	12.1	11.4	21.7
Jiangsu	556	66.0	45.1	42.8	14.6	15.8	24.4
Guangdong	541	65.0	43.1	29.4	8.5	6.1	14.7
Shaanxi	509	63.0	49.5	36.7	10.8	27.9	25.1
Shanxi	444	63.5	47.5	43.0	13.1	20.7	25.6
Liaoning	424	63.0	44.6	43.6	11.1	29.5	28.1
Fujian	401	64.0	48.1	27.7	8.5	12.2	16.1
Guangxi	375	67.0	41.1	37.3	9.9	11.7	19.6
Hubei	349	64.0	46.7	35.2	13.8	16.9	22.0
Gansu	336	63.0	44.6	45.8	14.3	28.3	29.5
Jilin	311	64.0	46.0	38.9	12.2	40.8	30.7
Heilongjiang	263	65.0	43.0	43.0	12.5	49.0	34.9
Chongqing	169	69.0	45.6	38.5	11.8	14.8	21.7
Guizhou	129	68.0	45.0	48.1	7.0	8.5	21.2
Tianjin	102	63.0	42.2	49.0	20.6	40.2	36.6
Qinghai	96	60.0	35.4	45.8	5.2	30.2	27.1
Xinjiang	73	63.0	43.8	43.8	16.4	42.5	34.2

Province	Participants	Median Age (years)	Male (%)	Hypertension (%)	Diabetes (%)	Cardiovascular (%)	Average Prevalence (%)
Shanghai	47	63.0	40.4	48.9	12.8	21.3	27.7
Beijing	34	69.5	41.2	58.8	23.5	32.4	38.2

We further investigated the associations between chronic diseases and the TyG index along with its related metrics (Fig 3). Due to the inherent connections in the definitions between the TyG index, conventional obesity indices, and their related parameters, they demonstrated strong intercorrelations. Although these indices showed statistically significant associations with multiple chronic diseases ($P < 0.05$), the correlation strengths remained relatively weak. This suggests that the development of chronic diseases may involve more complex mechanisms, requiring comprehensive consideration of various risk factors.

Fig 3. Pearson's correlation of TyG and related indicators with chronic diseases

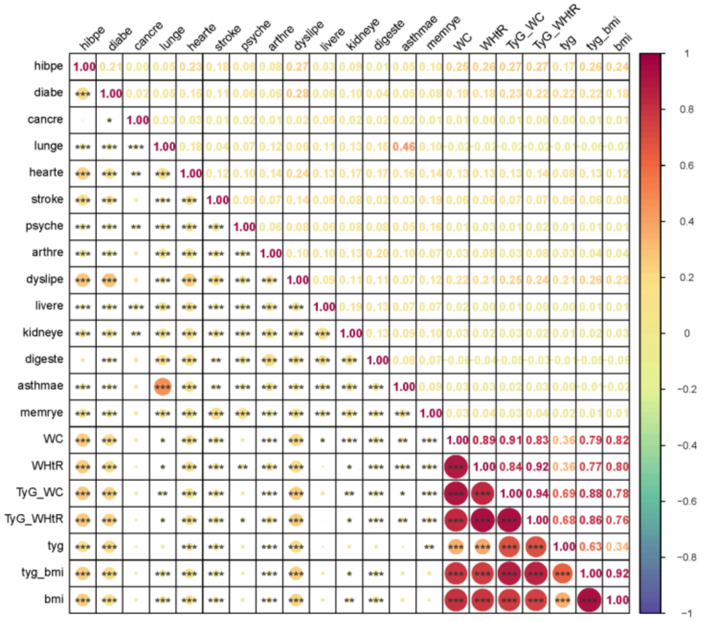

Relationships of TyG and related indicators with chronic diseases

We next analyzed the dose-response relationships between the TyG index and its related indicators with six chronic diseases (Fig 4). These indices showed positive dose-response relationships with the risks of diabetes, heart disease, dyslipidemia, hypertension, and stroke, though they exhibited different acceleration patterns. Some variables demonstrated exponential or near-linear positive correlation patterns, as seen in the dose-risk relationship between TyG and diabetes (Fig 4B), and between TyG-

WHtR and heart disease (Fig 4D). Other variables showed rapid growth within certain ranges, with the growth rate slowing down or even the risk potentially decreasing after reaching certain thresholds, as observed in the dose-risk relationship between TyG-BMI and both heart diseases and stroke (Fig 4A). Interestingly, some indices (TyG, TyG-WC, and WC) showed negative dose-response relationships with digestive system diseases, while others (TyG-BMI, TyG-WHtR, and WHtR) displayed an initial decrease followed by an increase, particularly showing higher risks at lower doses. These findings highlight the complexity and dynamic nature of these indicators in disease risk assessment, suggesting that these factors should be carefully considered when developing intervention strategies.

Fig 4. Dose-responsive relationship of the TyG and related indicators with the risk of chronic diseases

The study further employed a Cox proportional hazards model to analyze the relationships between different predictor variables and chronic diseases. For each metabolic indicator, participants were divided into terciles (three groups of equal size). Taking TyG as an example, participants were classified based on their TyG values into: Group 1 (TyG < 8.36), Group 2 ($8.36 \le$ TyG < 8.87), and Group 3 (TyG ≥ 8.87). The first tercile group

(Group 1) served as the reference group (Ref) for comparison with the other two tercile groups. Two adjusted models were simultaneously used to account for potential confounding factors. To address potential residual confounding, we conducted several sensitivity analyses with additional adjustments when data were available, and the results showed robustness. To verify whether the Cox models used in the study satisfied the proportional hazards assumption, tests for the proportional hazard assumption were conducted for all combinations of predictor variables and various chronic diseases (Table S1). The test results showed differences in the satisfaction of the proportional hazards assumption across different predictor variables and diseases. Specifically, TyG-BMI and BMI satisfied the proportional hazards assumption for most diseases. From the disease perspective, the risk predictions for stroke and digestive system diseases generally met the proportional hazards assumption, while predictions for dyslipidemia mostly violated this assumption. For models that violated the proportional hazards assumption, time-dependent covariate approaches were used for correction to ensure the reliability of the study results.

The study results confirmed the significant positive predictive effects of TyG and its related parameters on chronic disease risks across different tertiles (Table 4). The consistent outcomes from the two models enhance the robustness of our findings. For example, after additional adjustments

in Model 2, the hazard ratios of TyG tertile groups for hypertension remained nearly unchanged, indicating that the association between TyG and hypertension is independent of these sociodemographic and lifestyle factors. The same interpretation applies to all other metabolic indicators in the table. Compared to individuals in Tertile 1, those in Tertiles 2 and 3 exhibited a significant positive correlation between these indices and the risk of chronic diseases (adjusted HR > 1, and $P < 0.05$). Moreover, the HR values for various chronic diseases were higher in the higher TyG Tertile 3 compared to Tertile 2, indicating an increased risk of disease. Notably, in the context of diabetes, although the TyG index itself did not show a statistically significant direct association with diabetes risk in Tertile 2 ($P > 0.05$), but significant differences were observed in Tertile 3. Meanwhile, other indices such as TyG-BMI, WC, WHtR, TyG-WHtR, and BMI had HR values exceeding 1.5, especially in Tertile 3, these values rose above 4.0. This highlights the crucial role of these indices in diabetes risk assessment. However, there are exceptions. Specifically, in the case of digestive system diseases, most of these parameters showed no significant impact on the incidence of such diseases ($P > 0.05$). Only TyG-BMI, TyG-WC, and BMI were statistically significant ($P < 0.05$) in Tertile 3, and they were associated with a reduced risk of disease. Additionally, the associations between TyG and stroke risk, and between TyG-WC and the risks of heart disease and stroke, did not reach statistical significance (P >

0.05), suggesting that other unknown factors may be involved in regulating these diseases.

Table 4. Associations of TyG and related indicators with chronic diseases.

Index	Disease	Tertile1	Tertile2		Tertile3	
			**HR (95% CI)**	P	**HR (95% CI)**	P
TyG_Model1	hibpe	Ref	1.150 (1.070-1.237)	<0.001	1.324 (1.188-1.476)	<0.001
TyG_Model2	hibpe	Ref	1.152 (1.071-1.239)	<0.001	1.324 (1.188-1.476)	<0.001
TyG_Model1	diabe	Ref	1.142 (0.990-1.318)	0.068	1.399 (1.174-1.667)	<0.001
TyG_Model2	diabe	Ref	1.132 (0.981-1.306)	0.089	1.380 (1.157-1.645)	<0.001
TyG_Model1	hearte	Ref	1.142 (1.035-1.260)	0.008	1.180 (1.014-1.373)	0.032
TyG_Model2	hearte	Ref	1.130 (1.024-1.247)	0.015	1.157 (0.994-1.347)	0.059
TyG_Model1	stroke	Ref	1.047 (0.888-1.236)	0.586	1.126 (0.882-1.436)	0.341
TyG_Model2	stroke	Ref	1.049 (0.888-1.238)	0.575	1.132 (0.887-1.445)	0.319
TyG_Model1	dyslipe	Ref	1.194 (1.084-1.316)	<0.001	1.457 (1.275-1.666)	<0.001
TyG_Model2	dyslipe	Ref	1.179 (1.070-1.299)	<0.001	1.431 (1.251-1.636)	<0.001
TyG_Model1	digeste	Ref	0.985 (0.907-1.070)	0.719	0.988 (0.864-1.130)	0.862
TyG_Model2	digeste	Ref	0.987 (0.908-1.072)	0.752	0.992 (0.867-1.135)	0.903
TyG_BMI_Model1	hibpe	Ref	1.346 (1.263-1.434)	<0.001	1.941 (1.829-2.060)	<0.001
TyG_BMI_Model2	hibpe	Ref	1.346 (1.264-1.434)	<0.001	1.937 (1.824-2.057)	<0.001
TyG_BMI_Model1	diabe	Ref	1.832 (1.601-2.096)	<0.001	4.848 (4.301-5.463)	<0.001
TyG_BMI_Model2	diabe	Ref	1.800 (1.573-2.060)	<0.001	4.691 (4.160-5.289)	<0.001
TyG_BMI_Model1	hearte	Ref	1.238 (1.139-1.346)	<0.001	1.443 (1.332-1.564)	<0.001
TyG_BMI_Model2	hearte	Ref	1.213 (1.115-1.319)	<0.001	1.381 (1.274-1.498)	<0.001
TyG_BMI_Model1	stroke	Ref	1.264 (1.091-1.464)	0.002	1.806 (1.574-2.071)	<0.001
TyG_BMI_Model2	stroke	Ref	1.260 (1.088-1.460)	0.002	1.797 (1.565-2.064)	<0.001
TyG_BMI_Model1	dyslipe	Ref	1.529 (1.401-1.669)	<0.001	2.746 (2.535-2.973)	<0.001
TyG_BMI_Model2	dyslipe	Ref	1.491 (1.367-1.628)	<0.001	2.620 (2.419-2.839)	<0.001
TyG_BMI_Model1	digeste	Ref	0.952 (0.890-1.018)	0.151	0.909 (0.848-0.973)	0.006
TyG_BMI_Model2	digeste	Ref	0.959 (0.896-1.026)	0.222	0.924 (0.863-0.990)	0.024
WC_Model1	hibpe	Ref	1.215 (1.139-1.295)	<0.001	1.554 (1.460-1.655)	<0.001
WC_Model2	hibpe	Ref	1.220 (1.144-1.300)	<0.001	1.564 (1.469-1.666)	<0.001
WC_Model1	diabe	Ref	1.693 (1.478-1.939)	<0.001	4.090 (3.615-4.627)	<0.001

WC_Model2	diabe	Ref	1.675 (1.463-1.918)	<0.001	4.018 (3.551-4.547)	<0.001
WC_Model1	hearte	Ref	1.158 (1.064-1.260)	<0.001	1.248 (1.147-1.357)	<0.001
WC_Model2	hearte	Ref	1.142 (1.050-1.243)	<0.001	1.214 (1.115-1.320)	<0.001
WC_Model1	stroke	Ref	1.185 (1.022-1.374)	0.024	1.561 (1.353-1.801)	<0.001
WC_Model2	stroke	Ref	1.185 (1.022-1.375)	0.024	1.562 (1.353-1.804)	<0.001
WC_Model1	dyslipe	Ref	1.374 (1.258-1.500)	<0.001	2.178 (2.005-2.367)	<0.001
WC_Model2	dyslipe	Ref	1.353 (1.239-1.477)	<0.001	2.127 (1.957-2.311)	<0.001
WC_Model1	digeste	Ref	0.987 (0.922-1.055)	0.694	0.987 (0.922-1.057)	0.714
WC_Model2	digeste	Ref	0.991 (0.926-1.060)	0.793	0.998 (0.932-1.069)	0.963
TyG_WC_Model1	hibpe	Ref	1.086 (1.017-1.160)	0.014	1.139 (1.056-1.227)	<0.001
TyG_WC_Model2	hibpe	Ref	1.089 (1.020-1.164)	0.011	1.142 (1.060-1.231)	<0.001
TyG_WC_Model1	diabe	Ref	1.446(1.259-1.660)	<0.001	2.695 (2.343-3.100)	<0.001
TyG_WC_Model2	diabe	Ref	1.437 (1.252-1.650)	<0.001	2.679 (2.329-3.082)	<0.001
TyG_WC_Model1	hearte	Ref	1.072(0.982-1.169)	0.119	1.010 (0.914-1.117)	0.840
TyG_WC_Model2	hearte	Ref	1.063 (0.975-1.160)	0.168	0.997 (0.902-1.103)	0.958
TyG_WC_Model1	stroke	Ref	1.090 (0.936-1.269)	0.268	1.246 (1.052-1.477)	0.011
TyG_WC_Model2	stroke	Ref	1.090 (0.936-1.269)	0.267	1.247 (1.052-1.478)	0.011
TyG_WC_Model1	dyslipe	Ref	1.20 (1.099-1.316)	<0.001	1.497 (1.358-1.651)	<0.001
TyG_WC_Model2	dyslipe	Ref	1.192 (1.090-1.306)	<0.001	1.489 (1.351-1.643)	<0.001
TyG_WC_Model1	digeste	Ref	1.033 (0.964-1.107)	0.363	1.112 (1.028-1.202)	0.008
TyG_WC_Model2	digeste	Ref	1.034 (0.965-1.108)	0.339	1.116 (1.032-1.207)	0.006
WHtR_Model1	hibpe	Ref	1.321 (1.239-1.407)	<0.001	1.835 (1.728-1.949)	<0.001
WHtR_Model2	hibpe	Ref	1.321 (1.240-1.408)	<0.001	1.834 (1.727-1.948)	<0.001
WHtR_Model1	diabe	Ref	1.807 (1.579-2.067)	<0.001	4.668 (4.140-5.264)	<0.001
WHtR_Model2	diabe	Ref	1.776 (1.552-2.033)	<0.001	4.526 (4.011-5.106)	<0.001
WHtR_Model1	hearte	Ref	1.217 (1.120-1.324)	<0.001	1.383 (1.275-1.500)	<0.001
WHtR_Model2	hearte	Ref	1.193 (1.097-1.297)	<0.001	1.326 (1.221-1.439)	<0.001
WHtR_Model1	stroke	Ref	1.241 (1.072-1.438)	0.004	1.713 (1.492-1.966)	<0.001
WHtR_Model2	stroke	Ref	1.239(1.069-1.435)	0.004	1.709 (1.487-1.964)	<0.001
WHtR_Model1	dyslipe	Ref	1.505 (1.379-1.642)	<0.001	2.618 (2.417-2.837)	<0.001
WHtR_Model2	dyslipe	Ref	1.468 (1.346-1.602)	<0.001	2.504 (2.310-2.714)	<0.001
WHtR_Model1	digeste	Ref	0.972 (0.909-1.040)	0.412	0.955 (0.892-1.023)	0.189
WHtR_Model2	digeste	Ref	0.978(0.914-1.047)	0.522	0.970 (0.905-1.038)	0.376
TyG_WHtR_Model1	hibpe	Ref	1.265(1.187-1.348)	<0.001	1.642 (1.542-1.747)	<0.001
TyG_WHtR_Model2	hibpe	Ref	1.266 (1.188-1.349)	<0.001	1.640 (1.540-1.746)	<0.001
TyG_WHtR_Model1	diabe	Ref	1.736 (1.517-1.988)	<0.001	4.191 (3.705-4.740)	<0.001
TyG_WHtR_Model2	diabe	Ref	1.707 (1.491-1.954)	<0.001	4.060 (3.588-4.595)	<0.001
TyG_WHtR_Model1	hearte	Ref	1.184 (1.088-1.288)	<0.001	1.283 (1.177-1.399)	<0.001
TyG_WHtR_Model2	hearte	Ref	1.160 (1.066-1.263)	<0.001	1.230 (1.127-1.342)	<0.001

TyG_WHtR_Model1	stroke	Ref	1.199 (1.035-1.390)	0.016	1.563 (1.352-1.806)	<0.001
TyG_WHtR_Model2	stroke	Ref	1.197 (1.033-1.388)	0.017	1.560 (1.348-1.804)	<0.001
TyG_WHtR_Model1	dyslipe	Ref	1.448 (1.327-1.581)	<0.001	2.364 (2.175-2.568)	<0.001
TyG_WHtR_Model2	dyslipe	Ref	1.412 (1.294-1.542)	<0.001	2.256 (2.075-2.452)	<0.001
TyG_WHtR_Model1	digeste	Ref	1.004 (0.937-1.075)	0.919	1.038 (0.960-1.122)	0.346
TyG_WHtR_Model2	digeste	Ref	1.008 (0.940-1.080)	0.831	1.047 (0.969-1.133)	0.245
BMI_Model1	hibpe	Ref	1.351 (1.268-1.440)	<0.001	1.962 (1.848-2.082)	<0.001
BMI_Model2	hibpe	Ref	1.351 (1.268-1.440)	<0.001	1.958 (1.844-2.079)	<0.001
BMI_Model1	diabe	Ref	1.838 (1.607-2.104)	<0.001	4.897 (4.346-5.518)	<0.001
BMI_Model2	diabe	Ref	1.807 (1.579-2.068)	<0.001	4.741 (4.205-5.345)	<0.001
BMI_Model1	hearte	Ref	1.241 (1.142-1.350)	<0.001	1.452 (1.340-1.574)	<0.001
BMI_Model2	hearte	Ref	1.216 (1.118-1.322)	<0.001	1.391 (1.283-1.507)	<0.001
BMI_Model1	stroke	Ref	1.266 (1.093-1.466)	0.002	1.815 (1.583-2.080)	<0.001
BMI_Model2	stroke	Ref	1.262 (1.090-1.462)	0.002	1.807 (1.574-2.073)	<0.001
BMI_Model1	dyslipe	Ref	1.535 (1.406-1.675)	<0.001	2.774 (2.562-3.003)	<0.001
BMI_Model2	dyslipe	Ref	1.497 (1.372-1.634)	<0.001	2.648 (2.445-2.869)	<0.001
BMI_Model1	digeste	Ref	0.945 (0.888-1.016)	0.131	0.903 (0.845-0.965)	0.003
BMI_Model2	digeste	Ref	0.957 (0.895-1.024)	0.204	0.920 (0.861-0.984)	0.015

Model 1: age and gender were adjusted; Model 2: age, gender, marital status, residential area (urban/rural), education level, smoking status, and alcohol consumption were adjusted.

Predictive capacity comparison

Next, we evaluated the predictive performance of our model. the predictive performance of TyG and its related parameters for stroke, diabetes, dyslipidemia, heart disease, and hypertension gradually diminished over time (Fig 5A, B, D, E, and F) . These indices exhibited the highest AUC values in the first year. Notably, TyG-WC emerged as a critical predictor, with first-year AUC values reaching 0.678 (95% CI: 0.650-0.760) for stroke, 0.735 (95% CI: 0.720-0.749) for dyslipidemia, 0.664 (95% CI:

0.650-0.678) for heart disease, and 0.707 (95% CI: 0.697-0.716) for hypertension, highlighting its significant role in early disease warning. For diabetes, the overall predictive performance of the TyG index was particularly remarkable, with an initial AUC value as high as 0.771 (95% CI: 0.754-0.788). Despite a slight decline over the eight-year observation period, the AUC values remained stable at 0.731 (95% CI: 0.718-0.744), maintaining a high level of predictive accuracy, this further consolidates its position as the preferred indicator for long-term diabetes monitoring. Similarly, TyG-WC also performed well in long-term diabetes monitoring, with its C-index consistently above 0.7 throughout the 1 to 8-year period. Regarding hypertension, we observed that compared with other diseases, the predictive performance of TyG and its related parameters showed more pronounced decline over time, suggesting the need for more aggressive intervention measures to curb disease progression. Unlike the diminishing predictive performance seen with other chronic diseases, the predictive power for digestive diseases was relatively weak in the early stages but gradually strengthened over time. However, the overall AUC values remained relatively low (AUC < 0.6) (Fig 5C). Among the predictors, WC stood out as the best indicator, reaching its peak value in the seventh year, though the AUC value was only 0.579 (95% CI: 0.569-0.590).

Fig 5. Time-dependent predictive capacity of TyG and related

indicators for chronic diseases

Among all analyzed metabolic indicators, TyG and TyG-WC exhibited the best discriminative ability for diabetes prediction, with C-indexes of 0.723 (95% CI: 0.716–0.730) and 0.714 (95% CI: 0.707–0.721), respectively, both categorized as "good discrimination" ($C \geq 0.7$) (Table S2). For hypertension, cardiovascular diseases, stroke, and dyslipidemia, most indicators had C-indexes between 0.6 and 0.7, falling into the "acceptable discrimination" category. Notably, TyG-WC performed best in predicting hypertension (C=0.668, 95% CI: 0.661–0.676) and dyslipidemia (C=0.682, 95% CI: 0.675–0.689). All indicators showed weak performance in predicting digestive system diseases ($C < 0.6$), which aligns with expectations, as these metabolic metrics may have weaker associations with the pathological mechanisms of digestive disorders. Our models demonstrated varied performance in calibration slopes. The TyG-WHtR had the highest calibration slope (0.373) for predicting hypertension, while TyG had the lowest calibration slope (0.065) for predicting stroke. An ideal calibration slope should approach 1.0, and our results indicate a degree of overfitting. Hosmer-Lemeshow test results showed low p-values for most models, suggesting discrepancies between predicted probabilities and observed events. This may be attributed to the use of large cohort data, where even small deviations can lead to statistical significance due to the

large sample size.

Discussion

With the transformation of lifestyles and the rapid development of the economy, especially against the backdrop of the aging population in China, the main threats to human health have gradually shifted from traditional infectious diseases to chronic diseases. The year-on-year increase in incidence and mortality rates underscores the urgency of disease prevention, early diagnosis, and risk prediction. Previous studies have primarily focused on the associations between the TyG index and its related indices with single categories of diseases such as cardiovascular diseases, chronic kidney disease, or chronic lung disease, or used relatively single predictive indices that cannot comprehensively reflect the relationships with various diseases[23],[24],[27],[28]. In contrast, our study for the first time conducts a large-scale comprehensive analysis of the relationships between multiple chronic diseases and the TyG index and its related indices, aiming to comprehensively characterize the associations between TyG and its related indices with a variety of chronic diseases. Our research findings indicate that the TyG index is significantly correlated with obesity indices and TyG-modified parameters, and there is also a certain correlation with multiple chronic diseases. They exhibit a positive dose-response relationship with the risks of diabetes, heart disease, dyslipidemia, stroke,

and hypertension, while showing a negative dose-response relationship or a pattern of first decreasing and then increasing with digestive system diseases. Predictions of model performance show that the TyG index remains the best predictor for diabetes, while TyG-WC exhibits the best predictive performance for hypertension, dyslipidemia, stroke, and heart disease. Over time, the predictive performance of TyG and its related parameters gradually decreases for stroke, diabetes, dyslipidemia, heart disease, and hypertension, while gradually increasing for digestive system diseases—though the predictive value for the latter remains low. These findings are of great significance for the early identification and intervention of chronic disease risks.

The TyG index, obesity indices, and modified TyG indices have been widely used for risk prediction of various diseases. In our study, we combined the TyG index with obesity indices to comprehensively evaluate the associations between these metrics and various chronic diseases. According to our findings, correlation analyses showed relatively weak associations between these indices and multiple chronic diseases. However, in subsequent Cox proportional hazards regression analyses, significant impacts of these metrics on multiple chronic risks were still observed. Most of these parameters were associated with increased chronic risks, which is consistent with most previous studies[23],[27],[30].The results showed that

most dose-risk response curves exhibited an exponential or approximately linear positive correlation growth pattern, consistent with the findings of Dang[24] and Ren[31]. This pattern reflects the negative impacts of glucose-lipid metabolic disorders and obesity on physical health. However, there are also some high-risk "acceleration areas" or "deceleration areas" associated with the TyG index. This is not uncommon. In the study by Cui et al.[23], a J-shaped relationship was found between the modified TyG index and the incidence of coronary heart disease in the elderly, which they attributed to differences in study design between cohort and cross-sectional frameworks. Lyu et al.[32], discovered a significant inverse "J" shape relationship between TyG-BMI and all-cause mortality in heart failure, and a "U" shape association with heart failure readmission. Qiao et al.[33] suggested that the existence of a J-shaped pattern indicates potential harm to the body from extreme related indicators, and controlling these values around a threshold can help reduce the risk of disease. Differences in research design between cohort and cross-sectional frameworks may explain part of the discrepancies, while participants' age, gender, and region may also influence them. It may be necessary to account for such differences by examining the associations between various diseases and populations in different subgroups. In any case, maintaining a healthy range of TyG index and its related parameters can effectively reduce the risk of chronic diseases. This comprehensive approach to evaluating the

TyG index and its related indices provides valuable insights into the risk factors associated with chronic diseases. It emphasizes the importance of considering multiple parameters when assessing an individual's risk for developing these conditions.

Consistent with many studies, TyG demonstrated the best performance in the evaluation of diabetes model performance[34],[35], further consolidating its status as the preferred indicator for long-term diabetes monitoring. The good predictive efficacy of these findings may stem from the fact that the TyG index can more directly reflect the core pathophysiological mechanism of diabetes—glucose-lipid metabolic disorders[8]. However, based on a comprehensive assessment of model performance predictions, TyG-WC was the best predictive indicator for these diseases, particularly in stroke, heart disease, hypertension, and dyslipidemia. Although it was not the optimal index for diabetes and digestive system diseases, its performance remained strong. Therefore, this study suggests that TyG-WC can serve as a comprehensive evaluation parameter for chronic diseases. In addition to its good performance in this study, TyG-WC has also demonstrated strong predictive ability for disease risks in various other contexts, such as non-alcoholic fatty liver disease in Korean adults[36], diabetes in Japanese populations (especially short-term predictions of future diabetes)[37], and metabolic dysfunction-associated fatty liver disease in U.S. populations[38]. The superior performance of TyG-WC may

lie in its comprehensive reflection of glucose-lipid metabolism and visceral obesity, which more comprehensively covers the pathological basis of chronic diseases[22].

A rather amazing observation regarding the atypical presentation of digestive diseases was noted in our study. Digestive system diseases demonstrated distinct patterns that differed significantly from other chronic conditions, both in the dose-risk relationship analysis and in model performance evaluation. Digestive system diseases showed a negative dose-response relationship with TyG, TyG-WC, and WC, while demonstrating an initial decrease followed by an increase with TyG-BMI, TyG-WHtR, and WHtR - all of which were associated with higher disease risks at lower levels. Abnormally low TyG index, obesity indices and their modified variants may indicate severely insufficient energy intake or malnutrition, a metabolic state commonly observed in patients with digestive diseases that has been linked to worse disease prognosis and increased mortality risk[39],[40]. In the model performance evaluation, these indices showed lower AUC values for digestive diseases compared to other diseases, and interestingly, their AUC values increased over time. These findings suggest we may need to reassess and adjust our monitoring and intervention strategies for digestive system diseases to better align with their unique pathophysiological progression patterns.

The digestive system diseases encompass a broad range, and this risk curve and temporal prediction trend, which differ from other chronic diseases, can be attributed to various factors. These factors include differences in pathophysiological mechanisms, the impact of lifestyle and dietary habits, and even the influence of other underlying diseases more commonly found within this group of diseases. For example, some studies have found that higher TyG indices are positively correlated with the risk of gallstones in digestive system diseases and can serve as predictive indicators for gallstone risk[41],[42]. Modified TyG indices are positively associated with fatty liver risk[43]. Larger WC and WHtR are linked to increased incidence of digestive system cancers, with WC showing a stronger correlation with digestive system cancers[44]. However, an analysis by Liu et al.[45] found that BMI and TyG indices do not mediate the association between breakfast frequency and the risk of gastrointestinal cancer. Gao et al.'s research indicated that excessively high or low modified TyG indices may both increase the risk of digestive system diseases[46]. Together with our findings, these results suggest that these indicators are more strongly associated with metabolic disease risks. In the current study, the inclusion of a large number of metabolism-unrelated diseases may dilute the effects of TyG indices and related parameters. In some non-metabolic diseases, metabolic parameters reflecting certain energy reserves may even reduce disease risks. Therefore,

future large-sample studies are needed to analyze different subgroups

Long-term physiotherapy care for chronic diseases is a health, psychological, and economic problem that society cannot ignore, and identifying patients' risk factors for chronic diseases is conducive to timely measures for intervention and treatment. However, due to individual differences, variations in research designs, and the presence of various confounding factors, these risk assessments still face many challenges in clinical applications. Our study covered the period up to September 2020, encompassing the early stage of the COVID-19 pandemic in China. The longitudinal nature of the research enabled us to examine trends over time, which is particularly important for understanding the trajectory of health issues in the pre-pandemic and early pandemic phases. However, home quarantine during pandemic control measures may have reduced physical activity among older adults, limited chronic disease management (such as regular follow-ups for diabetes), and even caused many middle-aged and elderly individuals to delay disease diagnosis due to avoiding medical visits, potentially leading to reporting bias[47],[48]. Meanwhile, the generalizability of the study results in the "post-pandemic era" is limited. As our data only cover the early stage of the pandemic, the long-term health impacts after the pandemic have not been fully observed, and thus may not reflect the risks of metabolic disorders in older adults caused by the

pandemic. In addition, Due to the observational study design, we could not establish a causal relationship between the TyG index and its associated parameters and chronic diseases, while unmeasured confounders may have influenced the results due to their inherent nature. For instance, information on participants' occupations and medication use. As part of the study population had already retired, occupational information was not systematically collected. Occupational exposures may exert different effects on various diseases[49],[50], making it difficult to predict the associations of these indices. Additionally, participants' medication use was not recorded in the data, and drug use may interfere with the calculation of relevant indices and their associations with diseases. Although the shortcomings of our study and its practical clinical applications, which need to be further researched and confirmed, our results still provide guidance for the intervention and treatment of patients with chronic diseases.

Conclusion

This study provides significant insights into the relationship between the TyG index and its related parameters and chronic diseases in the elderly population. The findings reveal a positive dose-response relationship between the TyG index and its related parameters with the risk of diabetes, cardiovascular diseases, dyslipidemia, and stroke. Particularly, the TyG-

WC demonstrates strong predictive power in the early stages of these diseases, highlighting its utility as an early warning indicator. Interestingly, an inverse relationship was observed between the TyG index and digestive system diseases, suggesting distinct pathophysiological mechanisms at play. The results of our study underscore the complexity of chronic disease development and aid in the identification and risk stratification of diseases using the TyG index and its related parameters, emphasizing the necessity of adopting a multidimensional approach to health assessment.

Declarations

Ethics approval and consent to participate: not applicable.

Consent for publication: agree to publish

Institutional Review Board Statement: Not applicable.

Informed Consent Statement: Not applicable.

Data Availability Statement: All data relevant to this study can gain access to the database Dryad. https://figshare.com/s/22c974cd9c57ef4e2d3a

Conflicts of Interest: The authors declare no conflict of interest.

Funding: This research did not receive any specific grant from funding agencies in the public, commercial, or not-for-profit sectors.

Author Contributions: Hongli Liu, Xinmei Mao, Xuechen Wang conceptualized the study and were responsible for the overall coordination

of the research.Dan Xu, Ting Wen were in charge of data collection and preliminary analysis. Jipeng Li, Dan Xu, Ting Wen contributed to further data analysis and interpretation. Hongli Liu, Xinmei Mao, Xuechen Wang engaged in the in-depth discussion of the results and participated in the drafting of the manuscript. Jipeng Li critically revised the manuscript and was responsible for the final proofreading and submission. All authors have read and approved the final manuscript.

Acknowledgments: We express gratitude to the authors of previous studies for depositing their data in CHARLS. Additionally, we extend our appreciation to the researchers who generously shared their R script online, which greatly facilitated our data analysis process.

References

[1] About Chronic Diseases. [2024-08-27]. https://www.cdc.gov/chronic-disease/about/index.html.

[2] China N B O S. China Statistical Yearbook 2023[M]. Beijing: China Statistics Press, 2023.

[3] Foreman KJ, Marquez N, Dolgert A, Fukutaki K, Fullman N, McGaughey M, et al. Forecasting life expectancy, years of life lost, and all-cause and cause-specific mortality for 250 causes of death: reference and alternative scenarios for 2016-40 for 195 countries and territories. Lancet. 2018 Nov 10;392(10159):2052-2090. doi:

10.1016/S0140-6736(18)31694-5.

[4] National Health Commission of the People′s Republic of China Healthy China action (2019—2030 year). [2024-08-27]. http://www.nhc.gov.cn/guihuaxxs/s3585u/201907/e9275fb95d5b4295be8308415d4cd1b2.shtml.

[5] Zhou P, Wang S, Yan Y, Lu Q, Pei J, Guo W, et al. Association between chronic diseases and depression in the middle-aged and older adult Chinese population-a seven-year follow-up study based on CHARLS. Front Public Health. 2023 Jul 20;11:1176669. doi: 10.3389/fpubh.2023.1176669.

[6] Rahman MS, Hossain KS, Das S, Kundu S, Adegoke EO, Rahman MA, et al. Role of Insulin in Health and Disease: An Update. Int J Mol Sci. 2021 Jun 15;22(12):6403. doi: 10.3390/ijms22126403.

[7] Li M, Chi X, Wang Y, Setrerrahmane S, Xie W, Xu H. Trends in insulin resistance: insights into mechanisms and therapeutic strategy. Signal Transduct Target Ther. 2022 Jul 6;7(1):216. doi: 10.1038/s41392-022-01073-0.

[8] Lee SH, Park SY, Choi CS. Insulin Resistance: From Mechanisms to Therapeutic Strategies. Diabetes Metab J. 2022 Jan;46(1):15-37. doi: 10.4093/dmj.2021.0280.

[9] Zhao X, An X, Yang C, Sun W, Ji H, Lian F. The crucial role and mechanism of insulin resistance in metabolic disease. Front Endocrinol

(Lausanne). 2023 Mar 28;14:1149239. doi: 10.3389/fendo.2023.1149239.

[10] Spinelli R, Baboota RK, Gogg S, Beguinot F, Blüher M, Nerstedt A, et al. Increased cell senescence in human metabolic disorders. J Clin Invest. 2023 Jun 15;133(12):e169922. doi: 10.1172/JCI169922.

[11] Lin SY, Li WC, Yang TA, Chen YC, Yu W, Huang HY, et al. Optimal Threshold of Homeostasis Model Assessment of Insulin Resistance to Identify Metabolic Syndrome in a Chinese Population Aged 45 Years or Younger. Front Endocrinol (Lausanne). 2022 Jan 5;12:746747. doi: 10.3389/fendo.2021.746747.

[12] Lee J, Kim B, Kim W, Ahn C, Choi HY, Kim JG, et al. Lipid indices as simple and clinically useful surrogate markers for insulin resistance in the U.S. population. Sci Rep. 2021 Jan 27;11(1):2366. doi: 10.1038/s41598-021-82053-2.

[13] Tahapary DL, Pratisthita LB, Fitri NA, Marcella C, Wafa S, Kurniawan F, et al. Challenges in the diagnosis of insulin resistance: Focusing on the role of HOMA-IR and Tryglyceride/glucose index. Diabetes Metab Syndr. 2022 Aug;16(8):102581. doi: 10.1016/j.dsx.2022.102581.

[14] Adams-Huet B, Zubirán R, Remaley AT, Jialal I. The triglyceride-glucose index is superior to homeostasis model assessment of insulin resistance in predicting metabolic syndrome in an adult population in

the United States. J Clin Lipidol. 2024 Jul-Aug;18(4):e518-e524. doi: 10.1016/j.jacl.2024.04.130.

[15] Liu N, Liu C, Qu Z, Tan J. Association between the triglyceride-glucose index and chronic kidney disease in adults. Int Urol Nephrol. 2023 May;55(5):1279-1289. doi: 10.1007/s11255-022-03433-9.

[16] Wu TD, Fawzy A, Brigham E, McCormack MC, Rosas I, Villareal DT, et al. Association of Triglyceride-Glucose Index and Lung Health: A Population-Based Study. Chest. 2021 Sep;160(3):1026-1034. doi: 10.1016/j.chest.2021.03.056.

[17] Huang J, Rozi R, Ma J, Fu B, Lu Z, Liu J, et al. Association between higher triglyceride glucose index and increased risk of osteoarthritis: data from NHANES 2015-2020. BMC Public Health. 2024 Mar 11;24(1):758. doi: 10.1186/s12889-024-18272-9.

[18] Xue Y, Xu J, Li M, Gao Y. Potential screening indicators for early diagnosis of NAFLD/MAFLD and liver fibrosis: Triglyceride glucose index-related parameters. Front Endocrinol (Lausanne). 2022 Sep 2;13:951689. doi: 10.3389/fendo.2022.951689.

[19] Shi YY, Zheng R, Cai JJ, Qian SZ. The association between triglyceride glucose index and depression: data from NHANES 2005-2018. BMC Psychiatry. 2021 May 25;21(1):267. doi: 10.1186/s12888-021-03275-2.

[20] Guo K, Wang Q, Zhang L, et al. Relationship between changes in the

triglyceride glucose-body mass index and frail development trajectory and incidence in middle-aged and elderly individuals: a national cohort study. Cardiovasc Diabetol. 2024 Aug 16;23(1):304. doi: 10.1186/s12933-024-02373-1.

[21] Huang R, Wang Z, Chen J, Bao X, Xu N, Guo S, et al. Prognostic value of triglyceride glucose (TyG) index in patients with acute decompensated heart failure. Cardiovasc Diabetol. 2022 May 31;21(1):88. doi: 10.1186/s12933-022-01507-7.

[22] Zhang FL, Ren JX, Zhang P, Jin H, Qu Y, Yu Y, et al. Strong Association of Waist Circumference (WC), Body Mass Index (BMI), Waist-to-Height Ratio (WHtR), and Waist-to-Hip Ratio (WHR) with Diabetes: A Population-Based Cross-Sectional Study in Jilin Province, China. J Diabetes Res. 2021 May 14;2021:8812431. doi: 10.1155/2021/8812431.

[23] Cui C, Qi Y, Song J, Shang X, Han T, Han N, et al. Comparison of triglyceride glucose index and modified triglyceride glucose indices in prediction of cardiovascular diseases in middle aged and older Chinese adults. Cardiovasc Diabetol. 2024 May 29;23(1):185. doi: 10.1186/s12933-024-02278-z.

[24] Dang K, Wang X, Hu J, Zhang Y, Cheng L, Qi X, et al. The association between triglyceride-glucose index and its combination with obesity indicators and cardiovascular disease: NHANES 2003-2018.

Cardiovasc Diabetol. 2024 Jan 6;23(1):8. doi: 10.1186/s12933-023-02115-9.
[25] Huo RR, Liao Q, Zhai L, You XM, Zuo YL. Interacting and joint effects of triglyceride-glucose index (TyG) and body mass index on stroke risk and the mediating role of TyG in middle-aged and older Chinese adults: a nationwide prospective cohort study. Cardiovasc Diabetol. 2024 Jan 13;23(1):30. doi: 10.1186/s12933-024-02122-4.
[26] Huo RR, Zhai L, Liao Q, You XM. Changes in the triglyceride glucose-body mass index estimate the risk of stroke in middle-aged and older Chinese adults: a nationwide prospective cohort study. Cardiovasc Diabetol. 2023 Sep 16;22(1):254. doi: 10.1186/s12933-023-01983-5.
[27] Zhou WQ, Song X, Dong WH, Chen Z. Independent effect of the triglyceride-glucose index on all-cause mortality in critically ill patients with chronic obstructive pulmonary disease and asthma: A retrospective cohort study. Chron Respir Dis. 2024 Jan-Dec;21:14799731241245424. doi: 10.1177/14799731241245424.
[28] Kunutsor SK, Seidu S, Kurl S, Laukkanen JA. Baseline and usual triglyceride-glucose index and the risk of chronic kidney disease: a prospective cohort study. Geroscience. 2024 Jun;46(3):3035-3046. doi: 10.1007/s11357-023-01044-5.
[29] Zhou Y, Wang C, Che H, Cheng L, Zhu D, Rao C, et al. Association

between the triglyceride-glucose index and the risk of mortality among patients with chronic heart failure: results from a retrospective cohort study in China. Cardiovasc Diabetol. 2023 Jul 7;22(1):171. doi: 10.1186/s12933-023-01895-4..

[30] Chen T, Liu Y, Wu S, Long S, Feng L, Lu W, et al. Comparison of TyG and Newly TyG Related Indicators for Chronic Kidney Diseases Estimation in a Chinese Population. Diabetes Metab Syndr Obes. 2024 Aug 19;17:3063-3075. doi: 10.2147/DMSO.S469260.

[31] Ren Y, Cheng L, Qie R, Han M, Kong L, Yan W, et al. Dose-response association of Chinese visceral adiposity index with comorbidity of hypertension and diabetes mellitus among elderly people. Front Endocrinol (Lausanne). 2023 May 12;14:1187381. doi: 10.3389/fendo.2023.1187381.

[32] Lyu L, Wang X, Xu J, Liu Z, He Y, Zhu W, et al. Association between triglyceride glucose-body mass index and long-term adverse outcomes of heart failure patients with coronary heart disease. Cardiovasc Diabetol. 2024 May 9;23(1):162. doi: 10.1186/s12933-024-02213-2.

[33] Qiao Q, Liang K, Wang C, Wang L, Yan F, Chen L, et al. J-shaped association of the triglyceride glucose-body mass index with new-onset diabetes. Sci Rep. 2024 Jun 16;14(1):13882. doi: 10.1038/s41598-024-64784-0.

[34] Zhou Y, Yang G, Qu C, Chen J, Qian Y, Yuan L, et al. Predictive

performance of lipid parameters in identifying undiagnosed diabetes and prediabetes: a cross-sectional study in eastern China. BMC Endocr Disord. 2022 Mar 24;22(1):76. doi: 10.1186/s12902-022-00984-x.

[35] Du T, Yuan G, Zhang M, Zhou X, Sun X, Yu X. Clinical usefulness of lipid ratios, visceral adiposity indicators, and the triglycerides and glucose index as risk markers of insulin resistance. Cardiovasc Diabetol. 2014 Oct 20;13:146. doi: 10.1186/s12933-014-0146-3.

[36] Song S, Son DH, Baik SJ, Cho WJ, Lee YJ. Triglyceride Glucose-Waist Circumference (TyG-WC) Is a Reliable Marker to Predict Non-Alcoholic Fatty Liver Disease. Biomedicines. 2022 Sep 11;10(9):2251. doi: 10.3390/biomedicines10092251.

[37] Kuang M, Yang R, Huang X, Wang C, Sheng G, Xie G, et al. Assessing temporal differences in the predictive power of baseline TyG-related parameters for future diabetes: an analysis using time-dependent receiver operating characteristics. J Transl Med. 2023 May 4;21(1):299. doi: 10.1186/s12967-023-04159-7.

[38] Zou H, Ma X, Zhang F, Xie Y. Comparison of the diagnostic performance of twelve noninvasive scores of metabolic dysfunction-associated fatty liver disease. Lipids Health Dis. 2023 Sep 6;22(1):145. doi: 10.1186/s12944-023-01902-3.

[39] Russell LA, Balart MT, Serrano P, Armstrong D, Pinto-Sanchez MI. The complexities of approaching nutrition in inflammatory bowel

disease: current recommendations and future directions. Nutr Rev. 2022 Jan 10;80(2):215-229. doi: 10.1093/nutrit/nuab015.

[40] Siddiqui ATS, Parkash O, Hashmi SA. Malnutrition and liver disease in a developing country. World J Gastroenterol. 2021 Aug 14;27(30):4985-4998. doi: 10.3748/wjg.v27.i30.4985.

[41] Li H, Zhang C. Association between triglyceride-glucose index and gallstones: a cross-sectional study. Sci Rep. 2024 Aug 1;14(1):17778. doi: 10.1038/s41598-024-68841-6.

[42] Liao Q, Chen Y, Peng Q, Li C. Relationship between triglyceride-glucose index and gallstones risk: a population-based study. Front Endocrinol (Lausanne). 2024 Jul 11;15:1420999. doi: 10.3389/fendo.2024.1420999.

[43] Kim AH, Son DH, Lee YJ. Modified triglyceride-glucose index indices are reliable markers for predicting risk of metabolic dysfunction-associated fatty liver disease: a cross-sectional study. Front Endocrinol (Lausanne). 2024 Jan 22;14:1308265. doi: 10.3389/fendo.2023.1308265.

[44] Li X, Lian Y, Ping W, Wang K, Jiang L, Li S. Abdominal obesity and digestive system cancer: a systematic review and meta-analysis of prospective studies. BMC Public Health. 2023 Nov 27;23(1):2343. doi: 10.1186/s12889-023-17275-2.

[45] Liu T, Wang Y, Wang X, Liu C, Zhang Q, Song M, et al. Habitually

Skipping Breakfast Is Associated with the Risk of Gastrointestinal Cancers: Evidence from the Kailuan Cohort Study. J Gen Intern Med. 2023 Aug;38(11):2527-2536. doi: 10.1007/s11606-023-08094-7.

[46] Gao T, Ren M, Feng Y, Li Y, Zhang X, He S. Association of the atherogenic index of plasma and modified triglyceride-glucose indices with digestive diseases among middle-aged and older population in China. Lipids Health Dis. 2024 Nov 30;23(1):398. doi: 10.1186/s12944-024-02321-8.

[47] Lima-Martínez MM, Carrera Boada C, Madera-Silva MD, Marín W, Contreras M. COVID-19 and diabetes: A bidirectional relationship. Clin Investig Arterioscler. 2021 May-Jun;33(3):151-157. English, Spanish. doi: 10.1016/j.arteri.2020.10.001.

[48] Yang MC, Clayton C, Harris D, Pelletier C, Schmidt J, Zwicker JG, et al. A Qualitative Investigation on Chronic Disease Management and Prevention Among Older Adults During the COVID-19 Pandemic. Am J Health Promot. 2024 Mar;38(3):384-393. doi: 10.1177/08901171231218681.

[49] Dutta S, Gorain B, Choudhury H, Roychoudhury S, Sengupta P. Environmental and occupational exposure of metals and female reproductive health. Environ Sci Pollut Res Int. 2022 Sep;29(41):62067-62092. doi: 10.1007/s11356-021-16581-9.

[50] Spagnolo P, Ryerson CJ, Guler S, Feary J, Churg A, Fontenot AP, et

al. Occupational interstitial lung diseases. J Intern Med. 2023 Dec;294(6):798-815. doi: 10.1111/joim.13707.

Supporting information

Fig S1. Schematic diagram of data preprocessing

Table S1. Proportional Hazards Assumption

Predictor	Disease	Test Statistic	P Value	PH Assumption Met
TyG_WC	Diabetes	41.561	0	No
TyG_WC	Digestive Disease	0.731	0.3925	Yes
TyG_WC	Dyslipidemia	69.58	0	No
TyG_WC	Cardiovascular Disease	15.637	1e-04	No
TyG_WC	Hypertension	19.697	0	No
TyG_WC	Stroke	2.099	0.1474	Yes
TyG_WHtR	Diabetes	7.157	0.0075	No
TyG_WHtR	Digestive Disease	0.758	0.384	Yes
TyG_WHtR	Dyslipidemia	12.566	4e-04	No
TyG_WHtR	Cardiovascular Disease	3.983	0.046	No
TyG_WHtR	Hypertension	0.393	0.5307	Yes
TyG_WHtR	Stroke	0.168	0.6819	Yes

Predictor	Disease	Test Statistic	P Value	PH Assumption Met
WC	Diabetes	18.818	0	No
WC	Digestive Disease	0.17	0.6805	Yes
WC	Dyslipidemia	68.94	0	No
WC	Cardiovascular Disease	12.702	4e-04	No
WC	Hypertension	33.613	0	No
WC	Stroke	1.262	0.2613	Yes
WHtR	Diabetes	2.979	0.0843	Yes
WHtR	Digestive Disease	0.238	0.6259	Yes
WHtR	Dyslipidemia	9.042	0.0026	No
WHtR	Cardiovascular Disease	2.285	0.1306	Yes
WHtR	Hypertension	0.372	0.5419	Yes
WHtR	Stroke	0.014	0.9073	Yes
BMI	Diabetes	0.129	0.7191	Yes
BMI	Digestive Disease	4.638	0.0313	No
BMI	Dyslipidemia	1.09	0.2965	Yes
BMI	Cardiovascular Disease	0.208	0.6485	Yes
BMI	Hypertension	0.009	0.9227	Yes
BMI	Stroke	0.118	0.7307	Yes

Predictor	Disease	Test Statistic	P Value	PH Assumption Met
tyg	Diabetes	47.011	0	No
TyG	Digestive Disease	1.591	0.2071	Yes
TyG	Dyslipidemia	31.815	0	No
TyG	Cardiovascular Disease	8.863	0.0029	No
TyG	Hypertension	1.822	0.1771	Yes
TyG	Stroke	1.522	0.2173	Yes
TyG_BMI	Diabetes	0.004	0.9499	Yes
TyG_BMI	Digestive Disease	3.881	0.0488	No
TyG_BMI	Dyslipidemia	0.932	0.3344	Yes
TyG_BMI	Cardiovasculr Disease	1.545	0.2139	Yes
TyG_BMI	Hypertension	0.07	0.7906	Yes
TyG_BMI	Stroke	0.029	0.8658	Yes

Interpretation

Yes: The proportional hazards assumption is met. The effect of this predictor on hazard is constant over time.

NO:The proportional hazards assumption is violated. Consider time-dependent covariate or stratification.

Table S2. Model Calibration and Discrimination

Predictor	Disease	C Index (95% CI)	Calibration Slope	Calibration Intercept	Calibration Assessment	Discrimination Assessment
TyG	Hypertension	0.633 (0.625-0.64)	0.287	0.14	Poor calibration (p <= 0.05)	Acceptable discrimination (0.6 <= C < 0.7)
TyG	Diabetes	0.723 (0.716-0.73)	0.109	0.028	Poor calibration (p <= 0.05)	Good discrimination (0.7 <= C < 0.8)
TyG	Cardiovascular Disease	0.616 (0.608-0.623)	0.25	0.038	Poor calibration (p <= 0.05)	Acceptable discrimination (0.6 <= C < 0.7)
TyG	Stroke	0.643 (0.635-0.65)	0.065	0.006	Poor calibration (p <= 0.05)	Acceptable discrimination (0.6 <= C < 0.7)
TyG	Dyslipidemia	0.66 (0.652-0.667)	0.228	0.067	Poor calibration (p <= 0.05)	Acceptable discrimination (0.6 <= C < 0.7)
TyG	Digestive Disease	0.548 (0.54-0.555)	0.256	0.075	Poor calibration (p <= 0.05)	Poor discrimination (C < 0.6)
TyG_BMI	Hypertension	0.607 (0.6-0.615)	0.35	0.107	Poor calibration (p <= 0.05)	Acceptable discrimination (0.6 <= C < 0.7)
TyG_BMI	Diabetes	0.588 (0.58-0.595)	0.191	-0.001	Poor calibration (p <= 0.05)	Poor discrimination (C < 0.6)
TyG_BMI	Cardiovascular Disease	0.61 (0.602-0.617)	0.265	0.032	Poor calibration (p <= 0.05)	Acceptable discrimination (0.6 <= C < 0.7)
TyG_BMI	Stroke	0.624 (0.617-0.632)	0.072	0.005	Poor calibration (p <= 0.05)	Acceptable discrimination (0.6 <= C < 0.7)
TyG_BMI	Dyslipidemia	0.598 (0.591-0.606)	0.269	0.064	Poor calibration (p <= 0.05)	Poor discrimination (C < 0.6)
TyG_BMI	Digestive Disease	0.546 (0.538-0.554)	0.265	0.065	Poor calibration (p <= 0.05)	Poor discrimination (C < 0.6)
WC	Hypertension	0.664 (0.657-0.672)	0.301	0.113	Poor calibration (p <= 0.05)	Acceptable discrimination (0.6 <= C < 0.7)
WC	Diabetes	0.659 (0.652-0.667)	0.178	-0.012	Poor calibration (p <= 0.05)	Acceptable discrimination (0.6 <= C < 0.7)
WC	Cardiovascular Disease	0.629 (0.621-0.636)	0.25	0.032	Poor calibration (p <= 0.05)	Acceptable discrimination (0.6 <= C < 0.7)
WC	Stroke	0.642 (0.635-0.649)	0.068	0.002	Poor calibration (p <= 0.05)	Acceptable discrimination (0.6 <= C < 0.7)
WC	Dyslipidemia	0.661 (0.654-0.669)	0.251	0.049	Poor calibration (p <= 0.05)	Acceptable discrimination (0.6 <= C < 0.7)
WC	Digestive Disease	0.56 (0.552-0.568)	0.245	0.086	Poor calibration (p <= 0.05)	Poor discrimination (C < 0.6)
TyG_WC	Hypertension	0.668 (0.661-0.676)	0.279	0.127	Poor calibration (p <= 0.05)	Acceptable discrimination (0.6 <= C < 0.7)
TyG_WC	Diabetes	0.714 (0.707-0.721)	0.133	0.007	Poor calibration (p <= 0.05)	Good discrimination (0.7 <= C < 0.8)
TyG_WC	Cardiovascular Disease	0.63 (0.622-0.637)	0.241	0.038	Poor calibration (p <= 0.05)	Acceptable discrimination (0.6 <= C < 0.7)
TyG_WC	Stroke	0.651 (0.643-0.658)	0.063	0.005	Poor calibration (p <= 0.05)	Acceptable discrimination (0.6 <= C < 0.7)

TyG_WC	Dyslipidemia	0.682 (0.675-0.689)	0.222	0.06	Poor calibration (p <= 0.05)	Acceptable discrimination (0.6 <= C < 0.7)
TyG_WC	Digestive Disease	0.558 (0.55-0.566)	0.244	0.089	Poor calibration (p <= 0.05)	Poor discrimination (C < 0.6)
WHtR	Hypertension	0.635 (0.627-0.642)	0.372	0.067	Poor calibration (p <= 0.05)	Acceptable discrimination (0.6 <= C < 0.7)
WHtR	Diabetes	0.617 (0.609-0.624)	0.22	-0.034	Poor calibration (p <= 0.05)	Acceptable discrimination (0.6 <= C < 0.7)
WHtR	Cardiovascular Disease	0.618 (0.61-0.625)	0.259	0.029	Poor calibration (p <= 0.05)	Acceptable discrimination (0.6 <= C < 0.7)
WHtR	Stroke	0.635 (0.628-0.643)	0.072	0.001	Poor calibration (p <= 0.05)	Acceptable discrimination (0.6 <= C < 0.7)
WHtR	Dyslipidemia	0.623 (0.616-0.631)	0.307	0.023	Poor calibration (p <= 0.05)	Acceptable discrimination (0.6 <= C < 0.7)
WHtR	Digestive Disease	0.556 (0.549-0.564)	0.272	0.065	Poor calibration (p <= 0.05)	Poor discrimination (C < 0.6)
TyG_WHtR	Hypertension	0.647 (0.64-0.655)	0.373	0.06	Poor calibration (p <= 0.05)	Acceptable discrimination (0.6 <= C < 0.7)
TyG_WHtR	Diabetes	0.671 (0.664-0.678)	0.276	-0.087	Poor calibration (p <= 0.05)	Acceptable discrimination (0.6 <= C < 0.7)
TyG_WHtR	Cardiovascular Disease	0.622 (0.614-0.629)	0.259	0.027	Poor calibration (p <= 0.05)	Acceptable discrimination (0.6 <= C < 0.7)
TyG_WHtR	Stroke	0.644 (0.637-0.651)	0.073	-0.001	Poor calibration (p <= 0.05)	Acceptable discrimination (0.6 <= C < 0.7)
TyG_WHtR	Dyslipidemia	0.647 (0.639-0.654)	0.335	-0.004	Poor calibration (p <= 0.05)	Acceptable discrimination (0.6 <= C < 0.7)
TyG_WHtR	Digestive Disease	0.555 (0.548-0.563)	0.266	0.071	Poor calibration (p <= 0.05)	Poor discrimination (C < 0.6)
BMI	Hypertension	0.605 (0.597-0.612)	0.346	0.112	Poor calibration (p <= 0.05)	Acceptable discrimination (0.6 <= C < 0.7)
BMI	Diabetes	0.579 (0.571-0.586)	0.169	0.017	Poor calibration (p <= 0.05)	Poor discrimination (C < 0.6)
BMI	Cardiovascular Disease	0.609 (0.601-0.616)	0.264	0.033	Poor calibration (p <= 0.05)	Acceptable discrimination (0.6 <= C < 0.7)
BMI	Stroke	0.623 (0.616-0.631)	0.072	0.005	Poor calibration (p <= 0.05)	Acceptable discrimination (0.6 <= C < 0.7)
BMI	Dyslipidemia	0.593 (0.585-0.6)	0.261	0.071	Poor calibration (p <= 0.05)	Poor discrimination (C < 0.6)
BMI	Digestive Disease	0.546 (0.538-0.554)	0.265	0.065	Poor calibration (p <= 0.05)	Poor discrimination (C < 0.6)

PONE-D-25-03753

ORIGINALITY REPORT

19%	15%	17%	3%
SIMILARITY INDEX	INTERNET SOURCES	PUBLICATIONS	STUDENT PAPERS

PRIMARY SOURCES

1	www.frontiersin.org<br>Internet Source	2%
2	cardiab.biomedcentral.com<br>Internet Source	2%
3	www.ncbi.nlm.nih.gov<br>Internet Source	1%
4	www.researchsquare.com<br>Internet Source	<1%
5	pmc.ncbi.nlm.nih.gov<br>Internet Source	<1%
6	www.medrxiv.org<br>Internet Source	<1%
7	assets-eu.researchsquare.com<br>Internet Source	<1%
8	www.mdpi.com<br>Internet Source	<1%
9	www.pref.fukuoka.lg.jp<br>Internet Source	<1%
10	www.dovepress.com<br>Internet Source	<1%
11	pubmed.ncbi.nlm.nih.gov<br>Internet Source	<1%

12 Weipeng Li, Chaonan Shen, Weiya Kong, Xiaohui Zhou, Huimin Fan, Yuzhen Zhang, Zhongmin Liu, Liang Zheng. "Association between the triglyceride glucose-body mass index and future cardiovascular disease risk in a population with Cardiovascular-Kidney-Metabolic syndrome stage 0–3: a nationwide prospective cohort study", Cardiovascular Diabetology, 2024 <1%
Publication

13 old.actaitalica.it <1%
Internet Source

14 L F McKay, W G Brydon, M A Eastwood, J H Smith. "The influence of pentose on breath methane", The American Journal of Clinical Nutrition, 1981 <1%
Publication

15 journals.lww.com <1%
Internet Source

16 psc.ky.gov <1%
Internet Source

17 Duo Yang, Xiang Li, Chao Qu, Jiayi Yi, Hai Gao. "Comparison of triglyceride-glucose related indices in prediction of cardiometabolic disease incidence among US midlife women", Scientific Reports, 2025 <1%
Publication

18 Rong-Rui Huo, Qian Liao, Lu Zhai, Xue-Mei You, Yan-Li Zuo. "Interacting and joint effects of triglyceride-glucose index (TyG) and body mass index on stroke risk and the mediating role of TyG in middle-aged and older Chinese <1%

adults: a nationwide prospective cohort study", Cardiovascular Diabetology, 2024
Publication

19	Papanas, N.. "Gliclazide treatment lowers serum ICAM-1 levels in poorly controlled type 2 diabetic patients", Diabetes and Metabolism, 200609<br>Publication	<1%
20	Xuejie Wang, Ziqiu Wang, Wen Du, Xiaobo Ma, Jun Ma, Zijin Chen, Chenni Gao, Xiaonong Chen. "Predictive Value of TyG and TyG-BMI Indices for Non-Alcoholic Fatty Liver Disease in High-Altitude Regions of China: A Cross-Sectional Study", Journal of Clinical Medicine, 2024<br>Publication	<1%
21	academic.oup.com<br>Internet Source	<1%
22	Submitted to Tulane University<br>Student Paper	<1%
23	bmcpulmmed.biomedcentral.com<br>Internet Source	<1%
24	Xiangyu Chen, Xiaofu Du, Feng Lu, Jie Zhang, Chunxiao Xu, Mingbin Liang, Lijin Chen, Jieming Zhong. "The Association Between the Triglyceride–Glucose Index, Its Combination with the Body Roundness Index, and Chronic Kidney Disease in Patients with Type 2 Diabetes in Eastern China: A Preliminary Study", Nutrients, 2025<br>Publication	<1%
25	Hye-Min Park, Taehwa Han, Seok-Jae Heo, Yu-Jin Kwon. "Effectiveness of the triglyceride-	<1%

glucose index and triglyceride-glucose-related indices in predicting cardiovascular disease in middle-aged and older adults: A prospective cohort study", Journal of Clinical Lipidology, 2023
Publication

26	mdpi-res.com<br>Internet Source	<1 %
27	Jianan Hong, Ruiying Zhang, Haoxian Tang, Shiwan Wu, Yequn Chen, Xuerui Tan. "Comparison of triglyceride glucose index and modified triglyceride glucose indices in predicting cardiovascular diseases incidence among populations with cardiovascular-kidney-metabolic syndrome stages 0–3: a nationwide prospective cohort study", Cardiovascular Diabetology, 2025<br>Publication	<1 %
28	research-information.bris.ac.uk<br>Internet Source	<1 %
29	www.researchgate.net<br>Internet Source	<1 %
30	Liyan Fu, Qingxuan Xing, Xiaoqian Wang, Yaoyao Chen, Jingjing Kong, Jin Li, Baohong Yue. "Exploring the association between the TyG-WHtR index and the incidence of stroke in the obese population: based on NHANES data from 1998 to 2018", Journal of Stroke and Cerebrovascular Diseases, 2025<br>Publication	<1 %
31	Xiaoxiao Li, Bo Tang, Ouyang Yujie, Chuan Xu, Shuanghu Yuan. "Single-cell RNA Sequencing Analysis Reveals Cancer-associated Fibroblast	<1 %

Signature for Prediction of Clinical Outcomes and Immunotherapy in Gastric Cancer", Journal of Immunotherapy, 2024
Publication

32	Submitted to University of Edinburgh<br>Student Paper	<1 %
33	Xinyu Yu, Siqi Jiang, Zhiyu Chen, Keke Ren, Shan Li, Yetao Luo, Deqing Chen, Qinghua Zhao, Huanhuan Huang. "Association of triglyceride-glucose index and its combinations with sarcopenia among community-dwelling older adults: based on the Chongqing Aging and Sarcopenia Evaluation (CHASE) cohort", Frontiers in Physiology, 2025<br>Publication	<1 %
34	bmcpublichealth.biomedcentral.com<br>Internet Source	<1 %
35	Qiushi Ren, Yang Huang, Quan Liu, Tongxin Chu, Gang Li, Zhongkai Wu. "Association between triglyceride glucose-waist height ratio index and cardiovascular disease in middle-aged and older Chinese individuals: a nationwide cohort study", Cardiovascular Diabetology, 2024<br>Publication	<1 %
36	Yincai Lan, Guangzi Qi, Boya Li, Suren Sooranna, Dongshun Chen, Wenlian Rao, Yinxia Lin, Yaqin Pang. "Association of the Triglyceride-Glucose Index and Obesity Indicators with Multiple Chronic Diseases: A Longitudinal Cohort Study Based on CHARLS", Journal of Multidisciplinary Healthcare, 2025<br>Publication	<1 %

37 Yuansong Zhuang, Liliang Qiu, Dongjian Han, Zhentao Qiao et al. "The association between triglyceride-glucose index and related parameters and risk of cardiovascular disease in American adults under different glucose metabolic states", Diabetology & Metabolic Syndrome, 2024 <1%
Publication

38 assets.researchsquare.com <1%
Internet Source

39 Lei Li, Zichen Xu, Luqing Jiang, Lingdan Zhuang, Jianjun Huang, Daoqin Liu, Qiwen Wu. "Triglyceride-Glucose Index and Its Correlates: Associations with Serum Creatinine and Estimated Glomerular Filtration Rate in a Cross-Sectional Study from CHARLS 2011–2015", Metabolic Syndrome and Related Disorders, 2023 <1%
Publication

40 Qin Ling, Jiawei Chen, Xiao Liu, Yi Xu, Jianyong Ma, Peng Yu, Kai Zheng, Fuwei Liu, Jun Luo. "The triglyceride and glucose index and risk of nonalcoholic fatty liver disease: A dose–response meta-analysis", Frontiers in Endocrinology, 2023 <1%
Publication

41 Submitted to The University of Manchester <1%
Student Paper

42 Yan Chen, Kailing Xie, Yuanyuan Han, Haonan Ju, Jiaxi Sun, Xin Zhao. "The association between triglyceride-glucose index and its combination with systemic inflammation indicators and all-cause and cardiovascular <1%

mortality in the general US population: NHANES 1999–2018", Lipids in Health and Disease, 2024
Publication

43	Yaoyao Zhou, Yaqi Wang, Fangli Li, Yiming Shi, Taotao Wu, Yingshuai Li. "The relationship of serum klotho levels and triglyceride glucose index-related indicators", Lipids in Health and Disease, 2024<br>Publication	<1 %
44	Zihao Zhang, Lan Tan. "Association of insulin resistance-related indicators with cardiovascular disease in Chinese people with different glycemic states", Frontiers in Endocrinology, 2025<br>Publication	<1 %
45	Min Qi, Runjuan Qiao, Jian-Qing He. "The association between triglyceride-glucose index and related parameters and risk of tuberculosis infection in American adults under different glucose metabolic states: a cross-sectional study", BMC Public Health, 2025<br>Publication	<1 %
46	Weiya Li, Hongde Li, Shiqin Peng, Junli Li et al. "Prognostic effect of the TyG index on patients with severe aortic stenosis following transcatheter aortic valve replacement: a retrospective cohort study", Cardiovascular Diabetology, 2024<br>Publication	<1 %
47	Xin Yang, Huiting Rao, Yi Yuan, Nan Hu, Xinmei Zhang, Yingxin Zeng, Guodong Xia. "Correlation analysis of the triglyceride–	<1 %

glucose index and related parameters in metabolic dysfunction-associated fatty liver disease", Scientific Reports, 2025
Publication

48	medicaldialogues.in<br>Internet Source	<1 %
49	orcid.org<br>Internet Source	<1 %
50	researchonline.ljmu.ac.uk<br>Internet Source	<1 %
51	Verena Gounden, Sridevi Devaraj, Ishwarlal Jialal. "The role of the triglyceride-glucose index as a biomarker of cardio-metabolic syndromes", Lipids in Health and Disease, 2024<br>Publication	<1 %
52	lipidworld.biomedcentral.com<br>Internet Source	<1 %
53	oss.ejgo.net<br>Internet Source	<1 %
54	Jing-Yi Zhu, Mu-Yun Liu, Chang Sun. "Assessment of the triglyceride glucose index in adult patients with chronic diarrhea and constipation", World Journal of Clinical Cases, 2024<br>Publication	<1 %
55	Liyuan Zhuo, Mingxi Lai, Lulu Wan, Xuan Zhang, Ronglin Chen. "Cardiometabolic index and the risk of new-onset chronic diseases: results of a national prospective longitudinal study", Frontiers in Endocrinology, 2024<br>Publication	<1 %

56	Pierre Sie. "Reassessment of von Willebrand factor (VWF), VWF propeptide, factor VIII:C and plasminogen activator inhibitors 1 and 2 during normal pregnancy", British Journal of Haematology, 6/2003<br>Publication	<1 %
57	Victor R. Preedy. "Adhesion Molecules", CRC Press, 2019<br>Publication	<1 %
58	Yi Chen, Junyan Zhao, Yuchen Sun, Zhongjing Yang, Caizhe Yang, Di Zhu. "Association of the triglyceride glucose index with sudden cardiac death in the patients with diabetic foot ulcer", Diabetes Research and Clinical Practice, 2025<br>Publication	<1 %
59	Zewen Yang, Hongxia Gong, Fuqiang Kan, Ningning Ji. "Association between the triglyceride glucose (TyG) index and the risk of acute kidney injury in critically ill patients with heart failure: analysis of the MIMIC-IV database", Cardiovascular Diabetology, 2023<br>Publication	<1 %
60	Zijian Li, Shishu Yin, Gang Zhao, Xianglong Cao. "Association between sarcopenic obesity and osteoarthritis: The potential mediating role of insulin resistance", Experimental Gerontology, 2024<br>Publication	<1 %
61	pdfs.semanticscholar.org<br>Internet Source	<1 %
62	perpustakaan.poltekkes-malang.ac.id<br>Internet Source	<1 %

63 www.hstp.org.in <1 %
Internet Source

64 Bo Zhang, Yunjiao Zhang, Kexin Chang, Niuniu Hou et al. "Risk assessment model based on nucleotide metabolism-related genes highlights SLC27A2 as a potential therapeutic target in breast cancer", Journal of Cancer Research and Clinical Oncology, 2024 <1 %
Publication

65 Guoyu Wang, Cen Xu, Jian Wang, Zhimei Teng, Xiang Sha, Kai Xu, Ruzhu Wang, Li Zhu. "Triglyceride-glucose index is associated with microcirculatory resistance in patients with type 2 diabetes and STEMI undergoing primary PCI", Scientific Reports, 2025 <1 %
Publication

66 Wanchen Zhao, Hui Zhang, Zhen Li, Cong Ma, Xiaopeng Huo, Na Guo. "Association between the Alpha-1-acid glycoprotein concentrations and depression in US adult women: a cross-sectional study from NHANES 2021–2023", Frontiers in Psychiatry, 2025 <1 %
Publication

67 Yaning Feng, Liangying Yin, Haoran Huang, Yongheng Hu, Sitong Lin. "Assessing the impact of insulin resistance trajectories on cardiovascular disease risk using longitudinal targeted maximum likelihood estimation", Cardiovascular Diabetology, 2025 <1 %
Publication

68 Yaqi Wang, Bowen Wan, Xueying Xu, Qingyun Lv et al. "Association between self-neglect and incident cognitive impairment among Chinese <1 %

older adults and its subgroup differences", Archives of Gerontology and Geriatrics, 2025
Publication

69 Yu Yang, Shuqi Wang, Boying Jia, Shuchun Chen. "Association Between Triglyceride-Glucose Index and Lung Function Parameters in the General Population Undergoing Health Examinations", Diabetes, Metabolic Syndrome and Obesity, 2024
Publication
<1%

70 bmcendocrdisord.biomedcentral.com
Internet Source
<1%

71 bmcgeriatr.biomedcentral.com
Internet Source
<1%

72 daangene.com
Internet Source
<1%

73 Daisuke Yoshida, Shota Ikeda, Keisuke Shinohara, Masaya Kazurayama et al. "Triglyceride-Glucose Index Associated with Future Renal Function Decline in the General Population", Journal of General Internal Medicine, 2024
Publication
<1%

74 Jie Zhou, Hui Huang, Hao Huang, Jing Peng, Wenjuan Chen, Fuli Chen, Yijia Tang, Qiyong Li, Yan Xiong, Long Zhou. "Association of triglyceride-glucose index and its combination with adiposity-related indices with the incidence of myocardial infarction: a cohort study from the UK Biobank", International Journal of Obesity, 2024
Publication
<1%

75 Maosen Geng, Ke Zhang. "CRP-Albumin-Lymphocyte index (CALLYI) as a risk-predicting biomarker in association with osteoarthritis.", Springer Science and Business Media LLC, 2024
Publication <1%

76 Serena Low, Kay Chin Jonathon Khoo, Bastari Irwan, Chee Fang Sum, Tavintharan Subramaniam, Su Chi Lim, Tack Keong Michael Wong. "The Role of Triglyceride Glucose Index in Development of Type 2 Diabetes Mellitus", Diabetes Research and Clinical Practice, 2018
Publication <1%

77 Tiantian Gao, Mudan Ren, Yun Feng, Yarui Li, Xv Zhang, Shuixiang He. "Association of the atherogenic index of plasma and modified triglyceride-glucose indices with digestive diseases among middle-aged and older population in China", Lipids in Health and Disease, 2024
Publication <1%

78 cancer.jmir.org
Internet Source <1%

79 openveterinaryjournal.com
Internet Source <1%

80 www.acnut.com
Internet Source <1%

81 www.e-dmj.org
Internet Source <1%

82 www.jstage.jst.go.jp
Internet Source <1%

83	www.science.gov<br>Internet Source	<1%
84	www.springermedizin.de<br>Internet Source	<1%
85	"Annual Report on the Development of China's Special Economic Zones (2019)", Springer Science and Business Media LLC, 2022<br>Publication	<1%
86	Huoping Zheng, Meiling Xu, Jing Yang, Mingjun Xu. "Association between the triglyceride-glucose index and its combined obesity indicators and the risk of hypertension in middle-aged and older Chinese adults: A nationwide cross-sectional study", PLOS ONE, 2025<br>Publication	<1%
87	Jing Liu, Liying Guan, Meng Zhao, Qihang Li, An Song, Ling Gao, Haiyan Lin, Jiajun Zhao. "Association Between the Triglyceride–Glucose Index and Outcomes of Nonalcoholic Fatty Liver Disease: A Large-Scale Health Management Cohort Study", Diabetes, Metabolic Syndrome and Obesity: Targets and Therapy, 2021<br>Publication	<1%
88	Robbert Sanderman, Karen Morgan. "The Routledge International Handbook of Health Psychology - Global and Contemporary Issues", Routledge, 2025<br>Publication	<1%
89	Shan Li, Li An, Zhiqing Fu, Wei Zhang, Hongbin Liu. "Association between triglyceride-glucose	<1%

related indices and all-cause and cause-specific mortality in the general population: a cohort study", Cardiovascular Diabetology, 2024
Publication

90 Xue Xia, Shuohua Chen, Xue Tian, Qin Xu, Yijun Zhang, Xiaoli Zhang, Jing Li, Shouling Wu, Anxin Wang. "Association of triglyceride-glucose index and its related parameters with atherosclerotic cardiovascular disease: evidence from a 15-year follow-up of Kailuan cohort", Research Square Platform LLC, 2024 <1%
Publication

91 Ahindra Nag. "Synthesis and Applications of Carbohydrates, Lipids, and Steroids", CRC Press, 2025 <1%
Publication

92 Cancan Cui, Yitian Qi, Jiayin Song, Xinyun Shang et al. "Comparison of triglyceride glucose index and modified triglyceride glucose indices in prediction of cardiovascular diseases in middle aged and older Chinese adults", Cardiovascular Diabetology, 2024 <1%
Publication

93 Fucun Ma, Jiaying Hu, Zheng Gao, Xuekai Liu, Mingjian Bai, Guowei Liang. "Combined effect of triglyceride glucose-body mass index and hypertension on new-onset stroke: evidence from the China health and retirement longitudinal study", Frontiers in Public Health, 2024 <1%
Publication

94 Jie Xu, Dihui Cai, Yuheng Jiao, Yingying Liao, Yinyin Shen, Yunli Shen, Wei Han. "Insights <1%

into the complex relationship between triglyceride glucose-waist height ratio index, mean arterial pressure, and cardiovascular disease: a nationwide prospective cohort study", Cardiovascular Diabetology, 2025
Publication

95 Mohammad E. Khamseh, Mojtaba Malek, Soodeh Jahangiri, Sohrab Nobarani et al. "Insulin Resistance/Sensitivity Measures as Screening Indicators of Metabolic-Associated Fatty Liver Disease and Liver Fibrosis", Digestive Diseases and Sciences, 2024 <1%
Publication

96 Ruoyu Gou, Danni Dou, Mi Tian, Xiaoyu Chang, Yonggang Zhao, Xin Meng, Guanghua Li. "Association between triglyceride glucose index and hyperuricemia: a new evidence from China and the United States", Frontiers in Endocrinology, 2024 <1%
Publication

97 Xiuli Wang, Jie Liu, Ke Yu, Zhenhua Huang, Hanxiong Liu, Xiang Li. "Association between TyG-related parameters and NAFLD risk in Japanese non-obese population", Scientific Reports, 2025 <1%
Publication

98 Yanan Qiao, Yue Wang, Cheng Chen, Yueqing Huang, Chunhua Zhao. "Association between triglyceride-glucose (TyG) related indices and cardiovascular diseases and mortality among individuals with metabolic dysfunction-associated steatotic liver disease: a cohort study of UK Biobank", Cardiovascular Diabetology, 2025 <1%

Publication

99	Yue Zhang, Ruifeng Duan, Xin Chen, lijuan Wei. "Association between the triglyceride-glucose obesity-related index and risk of gallstone disease and first gallstone surgery: the NHANES 2017-2020", Springer Science and Business Media LLC, 2024<br>Publication	<1 %

Exclude quotes	Off	Exclude matches	Off
Exclude bibliography	On		
