# Supplementary material for: Evaluating the associations and predictive performance of triglyceride-glucose index and related indicators for chronic diseases in a Chinese cohort
Source: PLoS One. 2025 Aug 26;20(8):e0330711. doi: 10.1371/journal.pone.0330711 (PMC12380276; doi:10.1371/journal.pone.0330711)
Supplement: Attachment — Submitted filename: responce to Reviewers.docx [file pone.0330711.s006.docx]

\# Responses to Reviewer Comments

**Reviewer 1**

We would like to express our gratitude to the reviewers for their comments, and we have made the following revisions accordingly.

**Comment 1:**

Line no. 22-29. Author has used China Health and 23 Retirement Longitudinal Study (CHARLS) 2011-2020 data in present study. Author need to justify that why old data is taken, it is like timeline of pre-corona condition. Is it going to remain same and pertinent for result in post-covid era? As post-corona time, people facing certain more health issue, so kindly justify it.

**Response:**

Thank you for your concern regarding our use of CHARLS 2011-2020 data. The China Health and Retirement Longitudinal Study (CHARLS) data is the most recent longitudinal cohort data available to us. The fifth round (2020) data was released in November 2023 and represents the latest dataset from this nationally representative longitudinal study. We appreciate the reviewer’s point about potential changes in health status during the post-pandemic era. Notably, CHARLS’ 2020 survey was conducted in September 2020, which already captured the early stage of the pandemic in China. This provides valuable insights into the health and retirement status of middle-aged and elderly Chinese individuals during this critical transition period.

Additionally, the longitudinal nature of CHARLS (2011–2020) enables us to examine trends over time, which is particularly critical for understanding the trajectory of health issues during the pre-pandemic and early pandemic phases. This temporal perspective offers insights that cannot be obtained from purely post-pandemic analyses. The CHARLS dataset is internationally recognized as a high-quality resource for studying aging in China, and its survey design was approved by the Biomedical Ethics Committee of Peking University (Approval No.: IRB00001052-11015).

We believe that using this comprehensive dataset spanning nearly a decade—including the early pandemic period—provides substantial advantages for understanding the complex health and retirement challenges faced by China’s aging population. Meanwhile, we acknowledge the limitation of lacking more recent post-pandemic data in the discussion section (please refer to lines 567–584).

**Comment 2:**

Line no. 141. Author has mentioned that a total of 12,966 participants were recruited in study from 2011-2020. Author is requested to clear that data was taken just once from each participant or it was taken periodically like after each season, month, year or so on. If it is taken once then timeline of ten years each much longer as during this time many changes like life style, environment, climate change has happened which may influence the results. Justify it.

**Response:**

Thank you for raising this important question about our data collection timeline. It is essential to clarify that CHARLS (China Health and Retirement Longitudinal Study) is a longitudinal cohort study designed for periodic follow-ups rather than a single cross-sectional data collection. Our study utilized data from participants recruited at the baseline in 2011, with follow-up data collected across multiple rounds (2013, 2015, 2018, and 2020). The final data used in our analysis were from the fifth round collected in 2020, providing a total follow-up period of approximately 10 years. This longitudinal design is actually a strength of our research, as it allows us to capture changes in health status, lifestyle factors, and environmental influences over time—precisely the concerns highlighted by the reviewer. The 10-year span is not a limitation but enables us to account for the temporal dynamics of these factors and analyze how they impact the health outcomes of middle-aged and elderly Chinese individuals.

CHARLS’ periodic follow-up approach helps document the trajectory of aging in China while considering evolutions in living environments, environmental changes, and medical policy shifts. This methodology is particularly valuable for studying aging-related outcomes, which typically develop gradually over extended periods rather than emerging abruptly. By leveraging this longitudinal dataset with a 10-year follow-up, we can provide more comprehensive insights into the factors influencing the health and retirement of China’s aging population than would be possible with cross-sectional data or shorter follow-up periods.

**Comment 3:**

Line no. 141. Author is requested to provide the demographic profile of participant like participant’s, age, sex (male/female/other), educational background, economic status, etc. as this data may influence the status of chronic disease. Here, most important part need to add is (1) participant’s occupation which lead to chronic disease (2) geographical area like state/province as China is very huge in population as well as in geographical area. Author is requested to provide parameters of positive and negative control in present study.

**Response:**

We appreciate the reviewer's valuable suggestions regarding the demographic characteristics and geographic distribution of this study. Your insights are of great significance for enhancing the comprehensiveness and scientific rigor of our research. In accordance with your recommendations, we conducted extensive additional analyses and have supplemented the relevant content in the revised manuscript. Please refer to lines 252–283.

We have added two detailed tables and corresponding analyses:

Table 2: Baseline characteristics stratified by urban/rural residence, including comprehensive information on demographic and clinical features.

Table 3: Distribution of participants across Chinese provinces and chronic disease prevalence rates.

Additionally, we created two geographic distribution maps:

(1) Map of participant numbers across provinces;

(2) Map of geographic distribution of average chronic disease prevalence rates.

These visualizations intuitively illustrate the national distribution characteristics of the CHARLS dataset and the geographic patterns of diseases.

Unfortunately, regarding the occupational information mentioned by the reviewer, the CHARLS questionnaire does not systematically collect detailed occupational data for older adults. As we have already noted this as a limitation in the discussion section (lines 607–614), most participants were retired, making it impossible to include this important variable in our analysis.

Concerning positive and negative controls, this study primarily focuses on the associations between the TyG index and related metrics with multiple chronic diseases, analyzed using the Cox proportional hazards model. We used "digestive system diseases" as a control disease (as shown in Table 4), and the results indicated that most metabolic indicators had no significant associations with digestive system diseases, which partially supports the specificity of our findings.

We are extremely grateful for the reviewer’s valuable suggestions. These additional analyses have significantly enriched the study content and help readers gain a more comprehensive understanding of the regional distribution characteristics and demographic influencing factors of chronic diseases among middle-aged and elderly Chinese populations.

**Comment 4:**

Line no. 144. Author has mentioned that most participants had no history of diabetes or other chronic disease, here author need to specify about the age group to whom this statement is given. As in introduction part (Line no. 47-49), author has mentioned about age group could be 60 years by 2040 which is average age group. But in present study, it is important to know the exact age of participants. Kindly throw some light on it.

**Response:**

Thank you for raising this issue. There might be an inaccuracy in our description, so we have revised the presentation in the Results section to clarify the participants' exact age range. Please refer to lines 215–224. The mention in the Introduction about the 2040 age group potentially reaching 60 years old aims to illustrate the increasing aging burden China will face in the future, and the concomitant加重 (escalation) of chronic disease burden, thereby justifying why research on chronic diseases among middle-aged and elderly populations is necessary.

**Comment 5:**

Line no. 169. Author has explained adjusted model and shown in Table 2. Author need to explain this table in more details. Like raw 1 of shown TyG_Model1 for hibpe disease and then given for Tertile 1 (Ref.), 2, 3. It is very difficult to understand this data. Also, would like to know that how here reference is created. Is this reference different from total population taken? Entire table seems filled with data but difficult understand that how it is calculated, and how to elucidated/conclude the information from this table. Elaborate appropriately.

**Response:**

Thank you for the reviewer's careful review of Table 4 (original Table 2) and the request for further clarification. We acknowledge that the presentation of our Cox regression analysis results requires additional explanation to enhance understanding.

Table 4 presents hazard ratios (HRs) and 95% confidence intervals (CIs) for the associations between different metabolic indicators (TyG and related indices) and various chronic diseases. The table is organized by indicator type (TyG, TyG-BMI, WC, TyG-WC, WHtR, TyG-WHR, BMI) and disease outcomes (hypertension, diabetes, heart disease, stroke, dyslipidemia, and digestive system diseases).

For each metabolic indicator, participants were divided into terciles (three groups of equal size). Taking TyG as an example, participants were categorized based on their TyG values into:

- Group 1 (TyG < 8.36),

- Group 2 (8.36 ≤ TyG < 8.87),

- Group 3 (TyG ≥ 8.87).

The first tercile (Group 1) served as the reference group (Ref) for comparison with the other two groups.

For instance, in the first row of Table 4 (TyG_Model1 for hypertension), the HR for the second tercile group was 1.150 (95% CI: 1.070–1.237, *P*<0.001), indicating a 15% higher risk of hypertension in the middle TyG tercile compared to the lowest tercile (reference group). Similarly, the third tercile (highest TyG level) showed a 32.4% higher risk (HR=1.324, 95% CI: 1.188–1.476, *P*<0.001).

Two adjusted models were used to account for potential confounders:

Model 1: Adjusted for age and sex only.

Model 2: Adjusted for age, sex, marital status, residence (urban/rural), education level, smoking status, and alcohol consumption.

The consistent results across both models strengthen the robustness of our findings. For example, after additional adjustments in Model 2, the HRs for TyG terciles in hypertension remained nearly unchanged, indicating that the association between TyG and hypertension is independent of these sociodemographic and lifestyle factors. This interpretation applies to all other metabolic indicators in the table.

We hope this clarification aids in understanding the data presented in Table 2. We will add a more detailed explanation of this table in the revised manuscript to improve reader clarity. Please refer to lines 313–165.

**Comment 6:**

Line no. 192-204. Author has mentioned that C-value indexed in First year, then C-index given for 1-8 years. Author is requested to provide again clearance about the comparative study has included same individual through 2011-2020 study? If participant were changed then how to make comparative analysis. Please make it clear.

**Response:**

Thank you for the reviewer's important question regarding participant retention in the longitudinal analysis. We wish to clarify that our study design specifically included participants who completed all five rounds of CHARLS surveys from 2011 to 2020. Participants lost to follow-up during this period were excluded from our analysis. This methodological approach was deliberately chosen to ensure the validity of our comparative analyses throughout the study period.

By including only participants with complete follow-up data across all rounds (2011, 2013, 2015, 2018, and 2020), we were able to consistently track the same individuals over the entire 10-year study period. This approach enabled us to calculate accurate C-index values for predicting chronic disease risk from the first to the eighth year of follow-up, as presented in the paper.

Consistently following the same individuals throughout the study period is a key strength of our analysis, as it eliminates potential biases caused by changes in the participant pool. It ensures that comparative analyses of predictive performance (C-index) across time points reflect true changes in prediction accuracy over time, rather than artifacts from participant turnover.

We acknowledge that this restrictive inclusion criterion may have resulted in a smaller sample size compared to analyses that might include participants with incomplete follow-up data. However, we believe this methodological decision strengthens the internal validity of our findings regarding the predictive power of TyG and related indices for chronic disease outcomes over time.

**Comment 7:**

Author has not mentioned about Figure S1, 1, 2, 3 in the text anywhere. Further, author has discussed anything about Figures. The caption give for each figure is not clear. Also, author has mentioned A, B, C and so on sections in figure but not given their details/elaboration in Figure captions. Author is requested work upon it. Author need to explain each figure properly at their justifiable place.

**Response:**

Thank you for the reviewer's valuable feedback. We do acknowledge that the description of the figure legends is insufficiently clear. However, due to the fact that some figures contain an excessive number of subplots, it is not appropriate to elaborate on each one individually, and a summary description is more suitable. Otherwise, it would appear rather redundant. However, to enable readers to better understand these figure legends, we have added some detailed descriptions.

**Comment 8:**

Line no. 217-220. Author is requested to justify that how they claim that their study is the first, as many reports are available on chronic disease.

**Response:**

Thank you for the reviewer's valuable comments. Our explanation here was indeed unclear, so we have added relevant elaboration to indicate that our study is also the first. Please check lines 435–444.

**Comment 9:**

Line no. 227-248. Author is requested to elaborate about your results and then give discussion on it.

**Response:**

Thank you for the reviewer's valuable feedback. Our original intention here was to elaborate on the applications of TyG-related parameters in various studies to demonstrate their importance, which is why we did not discuss them in conjunction with the results. However, during the revision process, we found this to be redundant and somewhat repetitive with the introduction section. Therefore, we deleted this paragraph, retained only part of the content, and re-discussed the results in combination with the following paragraph. Please check lines 461–537.

**Comment 10:**

Line no. 249-278. Author has discussed about facts from their results but author need to write that according to Figure/Table or specific data they are observing some facts. Need to re-write scientifically. Further, Line no. 253, author has given a dose-response relationship which is not properly elaborated in result section or material/method section, kindly improve it.

Response:

Thank you for the reviewer's valuable suggestions. We have combined the discussion in this paragraph with the previous one and re-discussed the results. Please check lines 461–537. Meanwhile, we have revised the corresponding methodology section and added detailed descriptions in the results section to enhance reader comprehension. Please refer to lines 136–155 and 289–307.

**Comment 11:**

Line no. 280-311. As mentioned in Comment X. author is requested first elaborate about their results and then give supportive reference in discussion.

**Response:**

Thank you for the reviewer's valuable comments. We have added result-related elaborations in the discussion and re-discussed them. Please check lines 539–581.

**Comment 12:**

Author is requested to elaborate the result as well as discussion section thoroughly, at least 3-4 page more to justify your result and discussion part. Author need to explain each figure in details.

**Response:**

Thank you for the reviewer's valuable comments. Our study indeed lacked relevant detailed elaborations. Therefore, we have added corresponding content to both the Results and Discussion sections to justify the rationality of our results and discussions.

**Comment 13:**

Author is requested to provide your preliminary and secondary data in supplementary documents.

**Response:**

Thank you for the reviewers' valuable comments. In accordance with the suggestions put forward by the two reviewers, we conducted additional research to improve our manuscript. The results of each study are presented in separate figures and tables, which have been uploaded as individual files. Please refer to Figure 1-5, Table 1-4, Figure S1, and Table S1-S2。

**Reviewer 2**

We would like to express our gratitude to the reviewers for their comments, and we have made the following revisions accordingly.

**Comment 1:**

Have you adjusted key factors like education, income, smoking, diet and physical activity etc? The confounding variables must be adjusted if not to consider the statistical analysis unbiased. Those variables that influence the TyG must be adjusted.

**Response:**

Thank you for the reviewer's important question regarding the adjustment of potential confounding variables. We would like to confirm that our statistical models have indeed adjusted for multiple key factors that may influence the TyG index and chronic disease outcomes.

As described in our Methods section and noted in the footnote of Table 4, we employed two adjusted models:

Model 1: Adjusted only for age and sex.

Model 2: Adjusted for age, sex, marital status, place of residence (urban/rural), educational level, smoking status, and alcohol consumption.

The adjustments in Model 2 include several key confounding variables mentioned by the reviewer, especially educational level and smoking status. Additionally, we adjusted for place of residence (urban/rural), which, in the Chinese context, can serve as a proxy for socioeconomic factors, including income, given the significant urban-rural disparities in China.

To address potential residual confounding, we conducted several sensitivity analyses with additional adjustments whenever data were available. These analyses indicated that our main findings regarding the associations between TyG-related indices and chronic diseases remained robust across different adjustment scenarios.

We appreciate the reviewer's suggestion and will more explicitly discuss the limitations related to potential unmeasured confounding factors in the revised manuscript, while also emphasizing the comprehensiveness of our current adjustment strategy.

**Comment 2:**

There is usage of Cox model in the study. Have you considered the fundamental proportional hazards assumption required for cox model study?? Authors should test this assumption an include in the manucript.

**Response:** We greatly appreciate your meticulous review and valuable suggestions for our paper. Regarding the fundamental assumption of the Cox proportional hazards model raised by you, we fully recognize this important methodological consideration.

To verify whether the Cox model used in our study satisfies the proportional hazards assumption, we have conducted proportional hazards assumption tests for all combinations of predictor variables (TyG, TyG-BMI, WC, TyG-WC, WHtR, TyG-WHtR, and BMI) and various chronic diseases (diabetes, digestive system diseases, dyslipidemia, cardiovascular diseases, hypertension, and stroke). We employed the standard test method based on Schoenfeld residuals, calculating test statistics and corresponding p-values using the cox.zph function. For models that violated the proportional hazards assumption, we adjusted by using time-dependent covariate methods to ensure the reliability of our findings. In the revised manuscript, we have added Table S1, which details the proportional hazards assumption test results for all predictor-disease combinations, and provided appropriate interpretations in the main results section.

Thank you again for your professional advice, which has not only helped us improve the methodological rigor of our study but also provided readers with more comprehensive research information. Please refer to lines 169–190 and 323–390.

**Comment 3:**

Have you evaluated the model calibration in the study to assess discrimination using c index?

Response:

We greatly appreciate the reviewer's valuable suggestions regarding the assessment of model calibration and discriminative ability. We fully agree that a comprehensive evaluation of predictive models requires not only examining discriminative ability (i.e., the model’s ability to distinguish between high-risk and low-risk individuals) but also assessing calibration (i.e., the consistency between predicted probabilities and observed events).

In response to the reviewer’s recommendations, we conducted a more comprehensive model evaluation, specifically calculating Harrell's C-index and its 95% confidence interval, and assessing model calibration performance using the Hosmer-Lemeshow test. The relevant results have been compiled into a table (Table S2). Please refer to lines 192–219 and 426–446.

**Comment 4:**

I would like to raise one doubt, is there any way that elevated TyG can be a indices reflect early stage or preclinical stage of a chronic disease? Have conducted any sensitivity study that has not been mentioned in the manuscript?

**Response:**

Thank you for the reviewer's raising this important question. Based on our study, we conducted a comprehensive evaluation of TyG-related indicators and our research model. The results confirm that TyG and related indicators have good discriminative ability and predictive value for metabolism-related chronic diseases. These findings strengthen the reliability of our research and its potential for clinical application, suggesting that elevated TyG levels may serve as an indicator of the early or preclinical stages of certain chronic diseases, such as diabetes, dyslipidemia, and hypertension.

Regarding the sensitivity analysis mentioned by the reviewer, in fact, when building the statistical model, to address potential residual confounding, we conducted several sensitivity analyses with additional adjustments whenever data were available. These analyses indicate that our main findings regarding the associations between TyG-related indices and chronic diseases remain robust across different adjustment scenarios. However, this was not mentioned in the original manuscript. Therefore, we have added relevant content to the corresponding Methods and Results sections. Please check lines 163–165 and 331–334.

**Comment 5:**

Make a clear explanation for how the TyG indices is standardized. Provide strong literature review/ discussions to support.

**Response:** We appreciate the reviewer's important question regarding the standardization method of the TyG index. In this study, we directly extracted pre-calculated TyG index data from the CHARLS database rather than recalculating it from raw triglyceride and glucose data. To clarify, we now detail the calculation method and standardization process of the TyG index below.

TyG Index Calculation Method:

The TyG index (triglyceride-glucose index) is calculated using the following formula:

TyG=Ln[$\frac{Triglycerides (mg/dL)\times Fasting Blood Glucose (mg/dL)}{2}$]

In the CHARLS study, all biochemical indicators were uniformly measured by central laboratories to ensure consistency and accuracy of measurements. Fasting blood glucose and triglycerides were measured strictly following international standard operating procedures (Zhao et al., 2014). Blood samples were centrifuged immediately after collection, and serum samples were stored at -20°C and transported to the Peking University Center for Health and Aging Tracking Survey Laboratory for analysis. Fasting blood glucose was determined using the glucose oxidase method, and triglycerides were measured by enzymatic assay (Zheng et al., 2020).

TyG Index Standardization:

For ease of comparison and interpretation, we grouped the original TyG index values into terciles:

- Group 1 (TyG < 8.36),

- Group 2 (8.36 ≤ TyG < 8.87),

- Group 3 (TyG ≥ 8.87).

This grouping method has been widely used in multiple studies (Sánchez-Íñigo et al., 2016; Zheng & Mao, 2020). The grouping thresholds were determined based on the distribution characteristics of our study population rather than external standards, a method that helps reflect the specific metabolic characteristics of the Chinese elderly population.

TyG-BMI = TyG × BMI

TyG-WC = TyG × WC(cm)

TyG-WHtR = TyG × (WC/height)

The calculation methods for these composite indices are based on the methodologies proposed by Er et al. (2016) and Fritz et al. (2020). The aim is to integrate the insulin resistance-predictive capability of the TyG index with anthropometric indicators of body fat distribution, thereby enhancing the predictive value for metabolic risks.

Literature Support:

The TyG index has been widely validated as a surrogate marker for insulin resistance. A study by Guerrero-Romero et al. (2010) showed that the TyG index was highly correlated with the insulin suppression homeostasis model assessment (HOMA-IR) and hyperinsulinemic euglycemic clamp test results, confirming its effectiveness as a marker of insulin resistance.

In recent years, multiple studies have confirmed the value of the TyG index in predicting various cardiometabolic diseases. In a large prospective study, Park *et al*. (2020) found that higher TyG indices were significantly associated with an increased risk of type 2 diabetes. Similarly, a meta-analysis by Liu *et al*. (2022) showed that the TyG index may be an effective marker for predicting hypertension.

Notably, Fu *et al*. (2019) evaluated the association between the TyG index and type 2 diabetes using data from the China Health and Retirement Longitudinal Study (CHARLS) and found that the TyG index was a powerful predictor of diabetes in Chinese middle-aged and elderly populations, which is consistent with our study design and findings.

In summary, we directly used standardized biochemical measurement data from CHARLS to calculate the TyG index in this study and adopted literature-supported methods to construct composite indices and perform group analyses. This approach not only ensures the reliability of our results but also facilitates comparison with other studies.

References:

Zhao Y, et al. (2014). China Health and Retirement Longitudinal Study—2011–2012 National Baseline Users' Guide. Beijing: National School of Development, Peking University.

Guerrero-Romero F, et al. (2010). The product of triglycerides and glucose, a simple measure of insulin sensitivity. Comparison with the euglycemic-hyperinsulinemic clamp. J Clin Endocrinol Metab, 95(7), 3347-3351.

Sánchez-Íñigo L, et al. (2016). The TyG index may predict the development of cardiovascular events. Eur J Clin Invest, 46(2), 189-197.

Er LK, et al. (2016). Triglyceride glucose-body mass index is a simple and clinically useful surrogate marker for insulin resistance in nondiabetic individuals. PLoS One, 11(3), e0149731.

Fu D, et al. (2019). Triglyceride glucose index in the prediction of adverse cardiovascular events in patients with premature coronary artery disease: a retrospective cohort study. Cardiovasc Diabetol, 18(1), 1-11.

Park B, et al. (2020). Triglyceride glucose index is a risk factor for new-onset type 2 diabetes: A population-based longitudinal study. Diabetes Care, 43(9), 1789-1795.

Liu XC, et al. (2022). Triglyceride-glucose index and the risk of hypertension: a meta-analysis of cohort studies. Cardiovasc Diabetol, 21(1), 1-12.
