# Supplementary material for: Evaluating the associations and predictive performance of triglyceride-glucose index and related indicators for chronic diseases in a Chinese cohort
Source: PLoS One. 2025 Aug 26;20(8):e0330711. doi: 10.1371/journal.pone.0330711 (PMC12380276; doi:10.1371/journal.pone.0330711)
Supplement: Attachment — Submitted filename: reponse to reviewers.docx [file pone.0330711.s008.docx]

\# Responses to Reviewer Comments

We sincerely appreciate the reviewers' recognition of our manuscript and their constructive comments for improving its quality. In response to the valuable suggestion, we have made the following revision:

**Comment:** In response to previous Comment 2 regarding the north-south prevalence disparity, author has mentioned that they had added explicit discussion of geographic distribution considerations in the Limitations section and asked to check lines 630-638. While observing the given lines in both cleared as well as check marked manuscript, the other information/conclusion is observed i.e. it is explicitly not clearly mentioned about north-south prevalence disparity. Author is requested to re-write the particular disparity in manuscript, and for easy understanding please mention the particular line no. either for cleared or check marked manuscript.

**Response:**

We apologize for the inadequacy of our explanation here. We have rephrased this part to accurately convey the issue of geographical differences and disease prevalence. Please check lines 630-640 in the document check marked manuscript.
